# Dissolved Organic Matter Contains Ketones Across a Wide Range of Molecular Formulas

**DOI:** 10.1021/acs.est.4c02593

**Published:** 2024-08-20

**Authors:** Nico Mitschke, Sahithya Phani Babu Vemulapalli, Thorsten Dittmar

**Affiliations:** † Institute for Chemistry and Biology of the Marine Environment (ICBM), School of Mathematics and Science, Carl von Ossietzky Universität Oldenburg, Ammerländer Heerstraße 114−118, Oldenburg 26129, Germany; ‡ Helmholtz Institute for Functional Marine Biodiversity (HIFMB) at the Carl von Ossietzky Universität Oldenburg, Ammerländer Heerstraße 231, Oldenburg 26129, Germany

**Keywords:** dissolved organic matter, reductive amination, isotopic labeling, ultrahigh-resolution mass spectrometry, FT-ICR-MS, ^15^N NMR spectroscopy, carbonyl, ketone

## Abstract

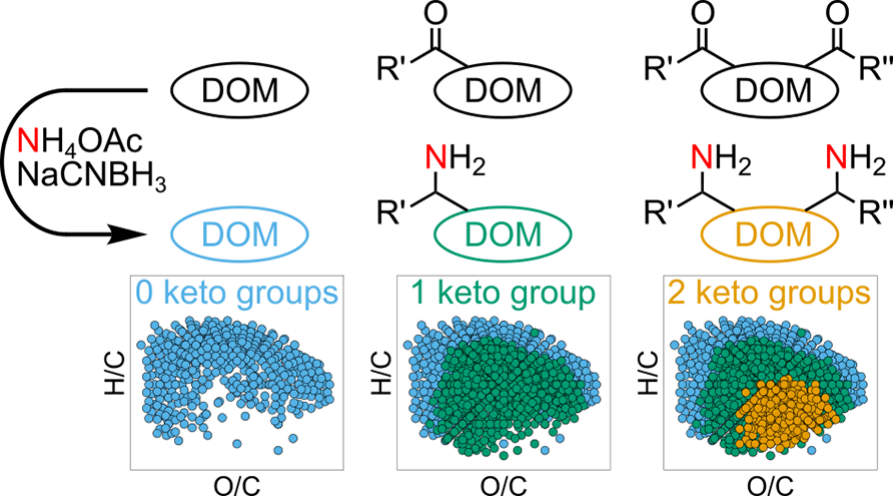

The carbonyl functionality of natural organic matter (NOM) is poorly constrained. Here, we treated Suwannee River NOM (SRNOM) with ammonium acetate and sodium cyanoborohydride to convert ketone-containing compounds by reductive amination to their corresponding primary amines. The total dissolved nitrogen content increased by up to 275% after amination. Up to 30% of the molecular formulas of SRNOM contained isomers with ketone functionalities as detected by ultrahigh-resolution mass spectrometry. Most of these isomers contained one or two keto groups. At least 3.5% of the oxygen in SRNOM was bound in ketone moieties. The conversion of reacted compounds increased linearly with O/H values of molecular formulas and was predictable from the elemental composition. The mean conversion rate of reacted compounds nearly followed a log-normal distribution. This distribution and the predictability of the proportion of ketone-containing isomers solely based on the molecular formula indicated a stochastic distribution of ketones across SRNOM compounds. We obtained isotopically labeled amines by using ^15^N-labeled ammonium acetate, facilitating the identification of reaction products and enabling NMR spectroscopic analysis. ^1^H,^15^N HSQC NMR experiments of derivatized samples containing less than 20 μg of nitrogen confirmed the predominant formation of primary amines, as expected from the reaction pathway.

## Abstract 2

We introduce a novel chemical derivatization method for the targeted analysis of carbonyl-containing compounds in dissolved organic matter, addressing a significant gap in its structural investigation.

## 1 Introduction

One of the largest carbon pools on Earth is dissolved organic matter (DOM), which contains ∼660 Pg of carbon in the ocean alone.^1^ DOM is operationally divided into a labile and a refractory fraction. Whereas the labile fraction is transformed within hours to days, the refractory fraction has accumulated over thousands of years. Structural insights that help to understand the cycling and role of DOM are currently extremely limited since only about 5% of DOM has been characterized on the level of molecular structures.^2^

In particular, the abundance of functional groups in DOM is poorly understood. The most abundant heteroatom in DOM is oxygen. Due to the wide range of structural moieties in which oxygen can occur, it is challenging to estimate the distribution of oxygen across different functional groups. The most important oxygen-containing functional groups in DOM are carboxyl groups, hydroxy groups, esters, ketones, aldehydes, ethers and (hemi)acetals/ketals, with carboxyl groups being the dominating functional group.^3^ The number of carboxyl groups can be estimated for individual molecular formulas from mass spectrometric fragmentation experiments and from O/H values of molecular formulas.^4^ Functional groups in natural organic matter (NOM) can also be selectively investigated by chemical derivatization or isotope exchange prior to mass spectrometric analysis. Examples for the analysis of carboxylic acids include their transformation to their corresponding methyl esters,^5,6^ H/D exchange to investigate labile hydrogens,^7,8^ and ^16^O/^18^O exchange to characterize nonlabile oxygens.^9^

Among the functional groups listed above, ketones and aldehydes are especially noteworthy because 1) they are more reactive than other common functional groups in NOM, 2) they strongly influence the optical properties of DOM (i.e., absorbance and fluorescence),^10^ which in turn affects water color and the absorption of wavelengths crucial for photosynthesis, and 3) they can serve as ligands to form metal complexes.^11^ Molecular-level characterization of ketones and aldehydes in NOM by derivatization approaches has been performed by reduction with sodium borohydride as well as sodium borodeuteride,^6,12,13^ by derivatization with *p*-toluenesulfonylhydrazine,^14,15^ by transforming them to hydrazones using Girard’s reagent,^16^ and by derivatization with *O*-(2,3,4,5,6-pentafluorobenzyl)hydroxylamine (PFBHA).^17,18^ In all cases, reaction products were analyzed by Fourier transform ion cyclotron mass spectrometry (FT-ICR-MS) or with an Orbitrap mass spectrometer.

These studies considerably advanced the field, but significant gaps in knowledge remain. Quantitative metrics such as conversions and mole percentages are lacking, as are estimates of the amount of oxygen bound as ketones and aldehydes and the proportion of isomers containing these structural features that can be derived from these metrics. Building on a previous study demonstrating that the amount of carboxyl groups for a given molecular formula depends solely on its elemental composition (i.e., the O/H values),^4^ we were curious if similar dependencies exist for the occurrence of ketones and aldehydes. For the enhanced structural characterization of NOM, NMR spectroscopy is of particular interest.^19^ However, derivatization products of NOM have never been comprehensively analyzed by combining ultrahigh-resolution mass spectrometry and NMR spectroscopy. To selectively analyze the derivatized functional groups by NMR spectroscopy, one must rely on an isotopic label (i.e., ^2^H for the above-mentioned studies), or on an NMR-active nucleus that is not abundant in DOM (e.g., ^19^F when using PFBHA). While the interpretation of ^2^H NMR spectra is often hindered by broad signals resulting from quadrupolar interactions (attributed to spin 1 of ^2^H), the fluorine atoms in PFBHA are at least six bonds away from the derivatized carbonyl carbon, which precludes the possibility of obtaining further insights from advanced NMR spectroscopic studies.

Another well-known reaction that specifically converts ketones and aldehydes is the reductive amination. This reaction proceeds in two steps: Initially, the ketone or aldehyde is transformed to an imine, which is subsequently reduced to the corresponding amine. If performed with ammonia or inorganic ammonium salts, the respective carbonyl functionalities are consequently transformed to their corresponding primary amines. Reductive aminations have been applied in synthetic chemistry for more than 130 years. Prominent examples are the Leuckart-Wallach,^20,21^ or the Eschweiler-Clarke reaction.^22,23^ In addition to the applicability of reductive aminations for the conversion of individual small organic compounds, reductive aminations have also been used for the derivatization of aldehyde functionalities in proteins.^24^ However, despite their long history, this type of reaction has not been investigated for the amination of complex natural organic mixtures.

A simple and convenient procedure for the reductive amination with ammonium has been proposed by Borch et al.,^25^ using 10 equivalents (equiv) of ammonium acetate (NH_4_OAc) and 0.7 equiv of lithium cyanoborohydride. This approach has several advantages: 1) the reaction is performed in methanol (MeOH) which is a commonly used solvent for extracting DOM from natural waters by solid-phase extraction (SPE), 2) NH_4_OAc can be exchanged by its ^15^N-labeled counterpart for stable isotope studies, and 3) the cyanoborohydride anion is a very mild reducing agent, selectively converting imines. Reductive aminations in general and the reaction proposed by Borch et al.^25^ in particular are highly selective for converting ketones and aldehydes, even in the presence of other functional groups. One notable example is the transformation of the complex fatty acid amide melonoside A that was selectively converted to the corresponding diamine in the presence of various coexisting functional groups (*cf*. Supporting Information, S1.2).^26^ Some functional groups such as acid chlorides and anhydrides may be converted to amides with ammonium acetate, or enamines may be reduced as their corresponding imines by NaBH_3_CN. However, these functional groups are unlikely to be major constituents of DOM.

The objective of this study was to narrow the existing gap in the characterization of ketone and aldehyde groups in DOM. To achieve this, we quantified the proportion of convertible compounds represented by distinct molecular formulas and the amount of oxygen bound in the form of these carbonyl functionalities. As a reference sample we chose Suwannee River NOM (SRNOM) as a widely used and commercially available representative for NOM in aquatic systems. SRNOM contains at most trace amounts of free aldehyde structural motifs as there are no pure aldehyde ^1^H NMR signals in SRNOM (*cf*. Figure S16, Supporting Information). Some aldehydes may be “masked” as acetals, e.g., in saccharides. However, for the sake of simplicity, we will refer in the following only to ketones as educts for the reductive amination. Since nitrogen is much less common in DOM compared to oxygen, we expected that an isotopic label is not necessarily needed to identify reaction products. However, the incorporation of ^15^N-labeled primary amines into DOM is prerequisite for ^15^N NMR spectroscopic studies. ^15^N NMR spectroscopy is an invaluable tool for the qualitative and quantitative analysis of nitrogen-containing molecules in NOM.^27,28^ We selectively analyzed ketone moieties as their corresponding ^15^N-labeled amines by NMR spectroscopy. Because of the low natural isotopic abundance of ^15^N (0.37%) we expect that NMR signals of isotopically labeled reaction products are easily distinguishable from background signals.

## 2 Materials and Methods

### 2.1 General Synthetic Aspects

All synthetic transformations were performed under inert conditions (argon atmosphere, exclusion of air and moisture) in anhydrous MeOH. Glassware and molecular sieve (3 Å) were precombusted (400 °C, 4 h) prior to use. All other material was washed by successively rinsing it with ultrapure water acidified with hydrochloric acid (∼8 mol/L) to pH 2 and ultrapure water.

### 2.2 Reductive Amination of Suwannee River Natural Organic Matter

Suwannee River natural organic matter (SRNOM, 2R101N^29^) was purchased from the International Humic Substances Society (IHSS) and dried for 16 h at 50 °C prior to use. Only limited information on the molecular-level structural composition of SRNOM is available, but the oxygen content was determined to be 41.5% (*w*/*w*).^30^ Thus, we calculated the equivalents of the reagents used for the reductive amination relative to this oxygen content, assuming conservatively that all available oxygen may be present in structural motifs amenable to the applied reaction conditions. For the derivatization, a solution of NH_4_OAc (7.99 mg, 0.104 mmol, 2 equiv; 40.0 mg, 0.519 mmol, 10 equiv; 200 mg, 2.59 mmol, 50 equiv) in MeOH (1 mL) was added to solid SRNOM (2.0 mg, corresponding to 0.0519 mmol oxygen) in 4 mL amber glass vials capped with a septum. Parallel experiments were performed with and without the addition of molecular sieve (3 Å, approximately 80 mg, corresponding to approximately 11 beads with 2 mm diameter). After a period of 30 min, NaBH_3_CN (1.30 mg, 0.0207 mmol, 0.4 equiv; 6.52 mg, 0.104 mmol, 2 equiv; 32.6 mg, 0.519 mmol, 10 equiv) was added to the mixture in one portion. After stirring for 48 or 168 h at ambient temperature, MeOH was evaporated under a stream of N_2_ at 50 °C and the residual was dissolved in 250 mL ultrapure water acidified with hydrochloric acid (∼8 mol/L) to pH 2 (caution: possible evolution of hydrogen cyanide). Samples were extracted by SPE using cartridges filled with styrene-divinylbenzene polymer (1 g, Bond Elut PPL, Agilent Technologies Inc.) and eluted with MeOH as described in detail by Dittmar et al.^31^ To determine the dissolved organic carbon (DOC) and total dissolved nitrogen (TDN) concentrations in SPE extracts, aliquots of 1 mL of each extract were dried in precombusted vials at 50 °C and redissolved in ultrapure water (10 mL) acidified with hydrochloric acid (∼8 mol/L) to pH 2. DOC and TDN measurements were performed with a TOC-V_CPH/CPN_ Total Organic Carbon Analyzer unit equipped with an ASI-V auto sampler (both Shimadzu Corp.).

### 2.3 FT-ICR-MS Measurements

Ultrahigh-resolution mass spectrometry measurements were performed with a solariX XR Fourier transform ion cyclotron mass spectrometer (Bruker Daltonics) equipped with a 15 T magnet and an Apollo II electrospray ionization source that was operated in negative ionization mode. Exact analytical conditions and further processing steps including molecular formula attribution with ICBM-OCEAN^32^ are described in more detail in the Supporting Information (S1.3). Exemplary mass spectra are shown in the Supporting Information (Figure S8–Figure S11).

### 2.4 NMR Measurements

NMR spectra were recorded in methanol-*d*_4_ (CD_3_OD) at 298 K using an Avance Neo 800 MHz (Bruker BioSpin) instrument equipped with a 5 mm BBO cryoprobe. Aliquots of samples corresponding to ∼0.3–0.4 mg DOC (∼0.01–0.02 mg TDN) were used. Methanol was evaporated under a stream of nitrogen and samples were redissolved in CD_3_OD (100 μL). To remove residual nondeuterated methanol, the samples were subjected to three cycles of evaporation and redissolving. Samples were finally dissolved in CD_3_OD (150 μL) and transferred into 3 mm NMR tubes. NMR data acquisition and processing were performed using TopSpin 4.3.0 (Bruker) and visualized using Sparky.^33^ One-dimensional (1D) ^1^H and ^15^N NMR spectra were obtained using the noesygppr1d and zgig30 pulse sequences, respectively, with standard acquisition parameters. Two-dimensional (2D) ^1^H,^15^N HSQC and ^1^H,^15^N HMBC NMR spectra were obtained using the hsqcedetgpsisp2.2 and the hmbcgpndqf pulse sequences, respectively. Experimental time was on average about 2 days per two-dimensional NMR experiment and sample. Experimental and processing parameters are described in more detail in the Supporting Information (S1.4).

### 2.5 Identification of Ketone-Containing Species

**Scheme 1:**
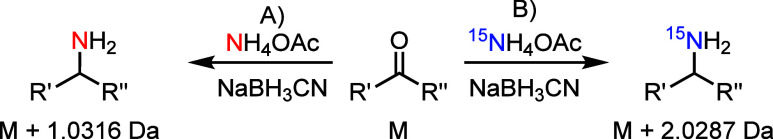
Reductive Amination of Ketones with (A) Unlabeled NH_4_OAc and NaBH_3_CN or (B) Isotopically Labeled ^15^NH_4_OAc and NaBH_3_CN

The reductive amination of a single ketone moiety increases the mass of a given molecular formula by 1.0316 Da when performing the reaction with NH_4_OAc, or 2.0287 Da when performing the reaction with ^15^NH_4_OAc (Scheme 1). Thus, we searched for newly arising or intensity changing mass peaks after reductive amination with mass differences of n*1.0316 or n*2.0287 (*n* = 1, 2 or 3) with respect to mass peaks that were already detected prior to the amination (i.e., in the control samples). The search for reaction products was directly conducted on mass lists after removal of noise peaks in ICBM-OCEAN^32^ (i.e., peaks with intensities less than three times the method detection limit were removed). To link molecular properties such as molecular formulas to detected molecular ions containing keto groups, we subsequently assigned molecular formulas to the molecular ions with ICBM-OCEAN.^32^

We validated our approach with help of the well-established Kendrick mass defect on a subset of our data (KMD, *cf*. Supporting Information, S1.5 for details). A peak was only considered a product of the reductive amination, if the intensity ratio of the product and the educt peak in the derivatized sample exceeded the corresponding ratio in the control. We used the ratio of two peaks because ratios are much more reproducible than absolute signal intensities, which can fluctuate due to variations in sample concentrations and analytical variability.^34^ To assess the significance of differences, we restrained from using established thresholds of *p*-values, an approach that often leads to false conclusions.^35^ Instead, we allowed a range of uncertainty by multiplying the intensity ratios in the control with a fixed constant >1, which we refer to as the detection ratio *dr*. The detection ratio was individually optimized for each combination of control and samples in a way that the false positive rate was below 1% for all samples of a sample set. The number of false positives was determined by comparing the number of detections among controls with those in the derivatized samples. As a result, the number of product peaks in derivatized samples exceeded the number of falsely identified product peaks in controls by at least a factor of 100. In conventional statistical terms, this corresponds roughly to a significance level of *p* < 0.01 (<1%). This procedure is described in detail in the Supporting Information (S1.6). While this approach is effective, it is crucial to consider other potential sources of false positive detections. These could occur if the educt peak intensity decreases due to degradation or side reactions, while the product peak intensity remains constant. We have taken steps to address these potential pitfalls. The effect of degradation reactions was minimized by treating controls and derivatized samples identically, except for the addition of derivatization reagents. Side reactions can be largely excluded because we did not observe an increase in detections when higher amounts of reagents were used. Furthermore, we considered potential fluctuations in measurement sensitivity and concentrations to ensure reliable detection of reaction products as described in the Supporting Information (S1.7).

We only considered species with different numbers of detected ketones for further analysis, if all ketone-containing species were reliably detected according to S1.7. For instance, if a species with a single keto group was reliably detected, but a species with two keto groups was unreliably detected for a given molecular educt ion, this molecular ion was not considered for further analysis. However, these combinations of simultaneously reliably and unreliably detected products only occurred at a very small rate, typically less than 2% of all detections. We further calculated the conversion of all molecular ions that were shown to contain at least one reliably detected ketone species and determined the proportion of amination products relative to all detected molecular ions (S1.9 and S1.10, respectively). Because the proportion of amination products is product specific, we considered all reliably identified reaction products, even if another product was unreliably assigned to the corresponding educt ion.

## 3 Results and Discussion

### 3.1 Optimization of Reaction Conditions

Prior to the derivatization of SRNOM, we tested slightly modified reaction conditions chosen from the literature^36^ (10 equiv NH_4_OAc, 2 equiv NaBH_3_CN, reaction in MeOH for 48 h) by converting individual model compounds (*cf*. Supporting Information, S1.1 for details). Even sterically more demanding substrates such as cyclohexyl phenyl ketone were converted in high yields. For the derivatization of SRNOM, we tested different reaction conditions that are summarized in Table 1. SPE-DOC concentrations of samples that were treated with molecular sieve (entries 5 and 10) decreased compared to SPE-DOC concentrations of samples that were not treated with molecular sieve. SPE-DOC concentrations also decreased for reaction times of 168 h. The extraction efficiencies were 75 ± 11% and 59 ± 15% for reaction times of 48 and 168 h, respectively. The number of attributed molecular formulas increased for all samples that underwent reductive amination but was highest when using 10 equiv of NH_4_OAc and 2 equiv of NaBH_3_CN. Overall, one may expect that SPE recoveries may decline due to amination, because most amines are protonated in aqueous solution at pH 2. However, such effect was not detectable (Table 1).

**Table 1:** Reaction Conditions for the Reductive Amination of SRNOM with Unlabeled NH_4_OAc^e^

#	NH_4_OAc (equiv)	NaBH_3_CN (equiv)	MS^a^ (3 Å)	time (min)	SPE-DOC (μmol)	SPE-TDN (μmol)	EE^b^	Molecular formulas	IW^c^ N-containing molecular formulas [%]
1	0	0	n	48 h	59	1.1	88	1725	1.1
2	2	0.4	n	48 h	51	1.6	77	1842	11
3	10	2	n	48 h	53	2.6	79	2309	25
4	50	10	n	48 h	47	1.8	70	1946	13
5	10	2	y^d^	48 h	41	2.0	62	2192	26
6	0	0	n	168 h	31	0.7	46	1725	1.5
7	2	0.4	n	168 h	42	1.4	63	1861	13
8	10	2	n	168 h	42	2.5	63	2066	31
9	50	10	n	168 h	48	2.2	72	1951	17
10	10	2	y^d^	168 h	35	2.0	52	2145	32

a MS: molecular sieve.

b EE: extraction efficiency based on DOC, calculated with respect to a control containing 66.65 μmol DOC prior to extraction. This control was prepared by dissolving 2.02 mg SRNOM in anhydrous MeOH (2 mL), evaporating the MeOH under a stream of nitrogen at 50 °C and redissolving the residual in ultrapure water (250 mL) acidified with hydrochloric acid (∼8 mol/L) to pH 2.

c IW: intensity-weighted.

d Approximately 80 mg of molecular sieve was used.

e Equivalents (equiv) are based on the total oxygen content of SRNOM. Treatments 1–5 were performed in duplicates and treatments 6–10 in triplicates.

We further assessed the variance among all technical replicates by performing a principal coordinate analysis (PCoA) based on Bray-Curtis dissimilarities of all detected molecular formulas and their respective FT-ICR-MS signal intensities (Supporting Information, S2.2). The first two principal coordinates accounted for 85% of the variance among samples. Along PC1, the experiments were assigned to three distinct groups that are mainly characterized by the number of detected molecular formulas. Surprisingly, one group (orange ellipse) comprised reactions that were performed with highest and lowest amounts of reagents (2 or 50 equiv NH_4_OAc, 0.4 or 10 equiv NaBH_3_CN). The second group (blue ellipse) comprised the controls (reactions that were performed without the addition of NH_4_OAc and NaBH_3_CN) and the last group (green ellipse), which was most dissimilar to the controls, comprised reactions that were performed with medium amounts of reagents (10 equiv NH_4_OAc, 2 equiv NaBH_3_CN). This finding highlight that the use of medium amounts of reagents resulted in highest dissimilarities between samples and controls, consistent with the trends of general characteristics summarized in Table 1.

### 3.2 Incorporation of Nitrogen

The TDN concentration in the solid-phase extracted samples serves as a good indicator for the incorporation of nitrogen. Inorganic nitrogen compounds (i.e., ammonium or cyanide salts) are efficiently removed by the SPE.^31^ Residual NaBH_3_CN has been destroyed when acidifying the samples (hydrolysis to hydrogen gas, hydrogen cyanide and boric acid). In addition, if inorganic nitrogen contributed to the TDN, the TDN concentration of samples that were treated with 50 equiv NH_4_OAc and 10 equiv NaBH_3_CN should have had highest TDN values, which was not the case. TDN concentrations increased in all treatments except for the controls (Table 1, treatments 2–5 and 7–10, increase by 38–275%) and were highest for the reactions performed with 10 equiv NH_4_OAc, 2 equiv NaBH_3_CN and without molecular sieve, regardless of the reaction time. The incorporation of nitrogen is also well reflected in the intensity-weighted proportion of N-containing molecular formulas. This proportion increased about 20-fold from 1.1% to 11–26% for short reaction times (Table 1, entries 2–5) and from 1.5% to 13–32% for long reaction times (Table 1, entries 7–10), respectively.

### 3.3 Transformation of Ketone-Containing Compounds

**Figure 1:**
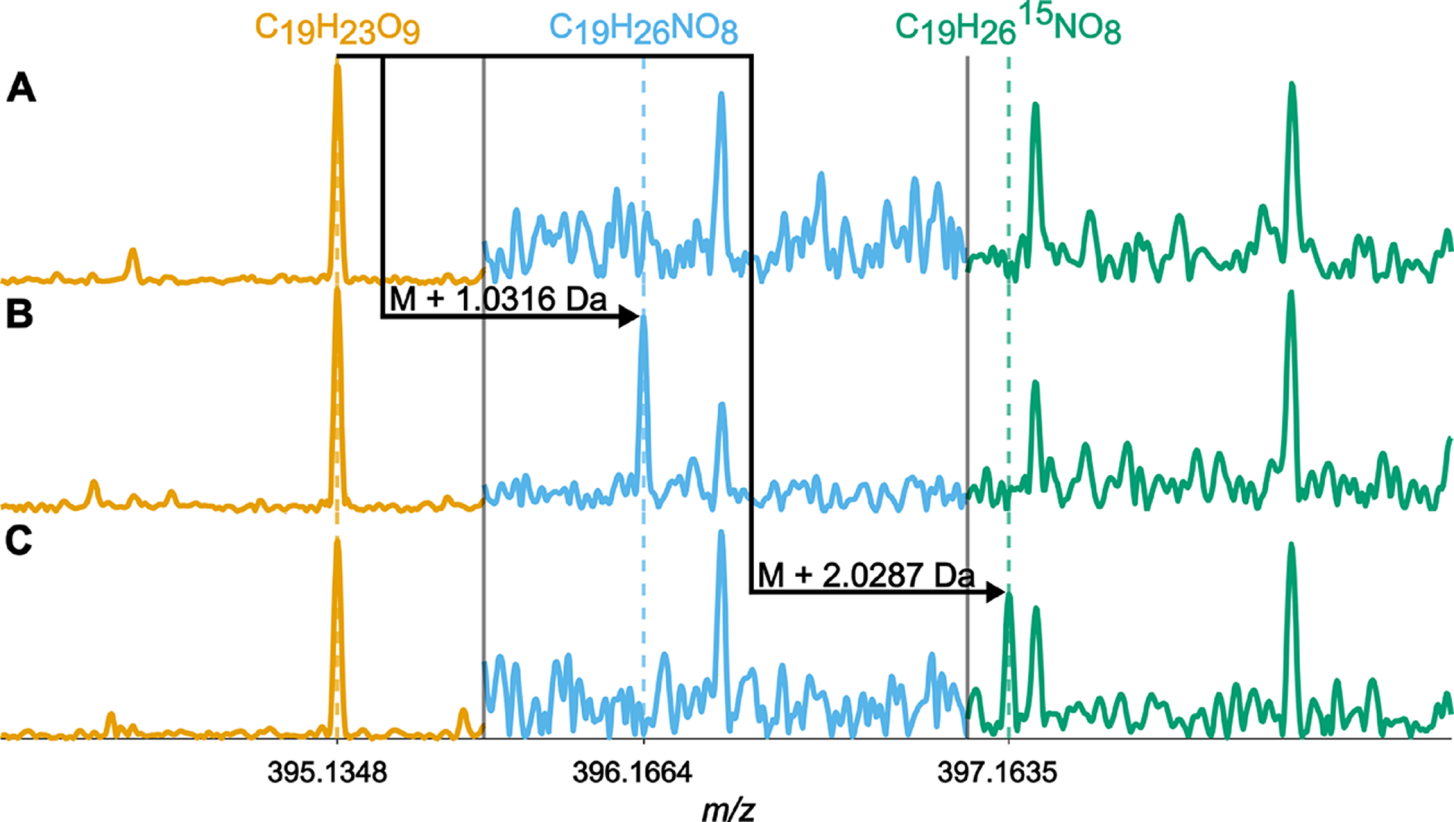
Selected mass spectral sections of SRNOM samples that were treated with A) no reagents (treatment 1, first technical replicate), B) NH_4_OAc/NaBH_3_CN (treatment 5, first technical replicate) and C) ^15^NH_4_OAc/NaBH_3_CN (treatment 14, second technical replicate).

Already the visual inspection of the ultrahigh-resolution mass spectra clearly showed that amination processes took place (Figure 1). For instance, no reaction product of C_19_H_23_O_9_^–^ (395.1348 Da) was detected in the controls (i.e., C_19_H_26_NO_8_^–^ at 396.1664 Da = 395.1348 Da + 1.0316 Da). However, the reaction product was detected at the predicted *m*/*z* value in all samples that were treated with NH_4_OAc and NaBH_3_CN. The optimized detection ratios (*dr*) were between 1.5 and 1.8 to obtain false positive rates below 1% for all samples. 86–97% of all the molecular formulas that were exclusively found in the samples treated with NH_4_OAc and NaBH_3_CN contained nitrogen. This observation indicates that almost no side reactions took place.

For a reaction time of 48 h, up to 1209 molecular ion masses representing compounds with at least one ketone functionality were detected. This is equivalent to 26–27% of all analyzed molecular ion masses. This finding is in overall agreement with previous studies addressing ketone-containing compounds in Suwannee River NOM by reduction with sodium borodeutride^12^ or derivatization with PFBHA.^17^ Most ions represented isomers with exactly one (877) or with simultaneously one and two (326) keto groups. Interestingly, most educt peaks were still present in the samples after derivatization, indicating an incomplete reaction or the presence of structurally very different isomers, including isomers without keto groups. Also, the simultaneous presence of one and two keto groups indicates isomeric diversity or incomplete reactions. Molecular ions that contained exclusively two keto groups (5 molecular ions) or simultaneously one, two and three keto groups (1 molecular ion) were hardly detected. No molecular ions were found containing exclusively three keto groups. Consequently, we did not search for more than three keto groups.

It should be noted that our routine occasionally assigns reaction products to more than one educt as depicted in more detail in the Supporting Information (S1.8). We counted exemplarily the number of reaction products that were assigned to two molecular ions representing species with exactly one or exactly one and two ketones. However, these “double detections” only occurred at a very small rate. Highest "double detections" were observed for a replicate of a reaction time of 168 h, where 18 of 739 reaction products that were assigned to molecular ions bearing one keto group were also assigned to molecular ions bearing exactly one and two keto groups. We deliberately decided to count these cases twice because usually no reliable statement can be made in these cases. The reaction products may be derived from compounds with two keto groups, from compounds with only one keto group, or simultaneously from both.

For reaction times of 168 h, a minor increase in the number of molecular ions bearing at least one ketone functionality was observed, compared to the shorter reaction time. From up to 1429 molecular ions containing at least one ketone, 940 contained exactly one, 451 contained one and two and 32 contained one, two and three ketones. Again, only a few molecular ions (6) contained exactly two, and none exclusively three ketones. Very similar to the shorter reaction time, 28–30% (and one outlier with 37%) of all detected molecular ions contained ketone functionalities. However, conversion rates differed sharply between treatments. While the mean conversion of ketone-containing species was up to 30% for reaction times of 48 h, it was up to 49% for reaction times of 168 h. The increase in conversion rate, but otherwise resembling patterns between treatments, indicate both incomplete reactions and isomeric diversity.

**Figure 2:**
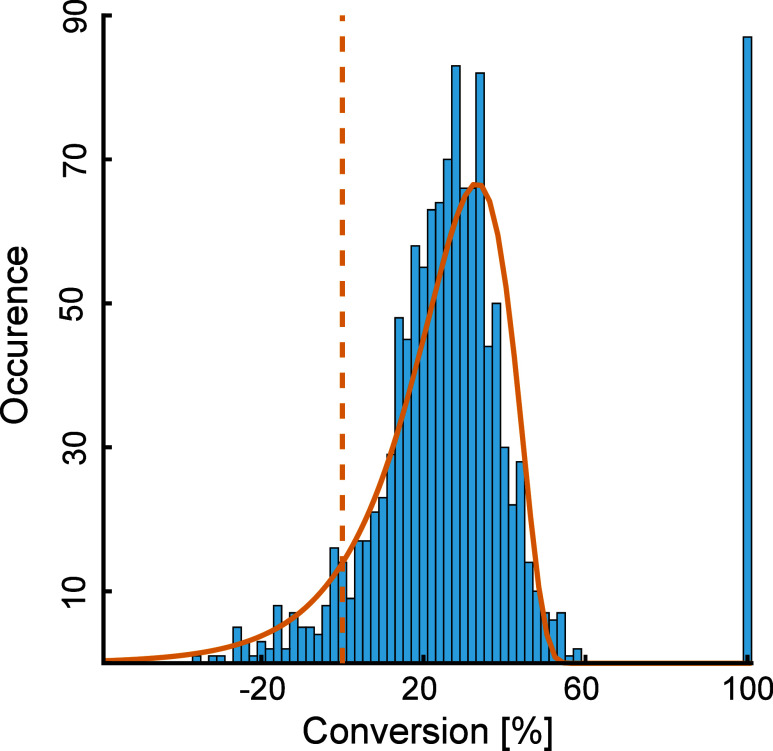
Distribution of conversions of molecular ions from the second technical replicate of treatment 3 that were reliably attributed (*dr* = 1.6) to contain at least one molecular structure with at least one ketone functionality. Due to inherent analytical variability of signal intensities, apparent negative conversions were occasionally observed (left to the orange dashed line). Educt compounds with an apparent conversion rate of 100% had low FT-ICR-MS signal intensities and likely fell below the detection limit after amination. The log-normal distribution was fitted to the data without using conversions of 100% and the direction of the *x*-axis was reversed to represent increasing conversions.

Remarkably, the conversions nearly followed a log-normal distribution with the exception that some molecular ions were converted to 100% (Figure 2). Complete conversion is probably an artifact because the respective molecular ions fell below the detection limit after amination. According to the central limit theorem, such a log-normal distribution could be the result of a stochastic reactivity of ketone functionalities across all DOM compounds.

In addition, we calculated the intensity increase of all molecular ions reliably identified as reaction products with respect to the total spectral intensity. Assuming similar ionization efficiencies of all molecular ions, this value allows a rough estimate of the mole percentage of ketone-containing compounds in SRNOM. For reaction times of 48 h, we found values up to 21% and for reaction times of 168 h values of up to 28%, implying that approximately every fourth molecule in SRNOM contains at least one ketone functionality.

**Figure 3:**
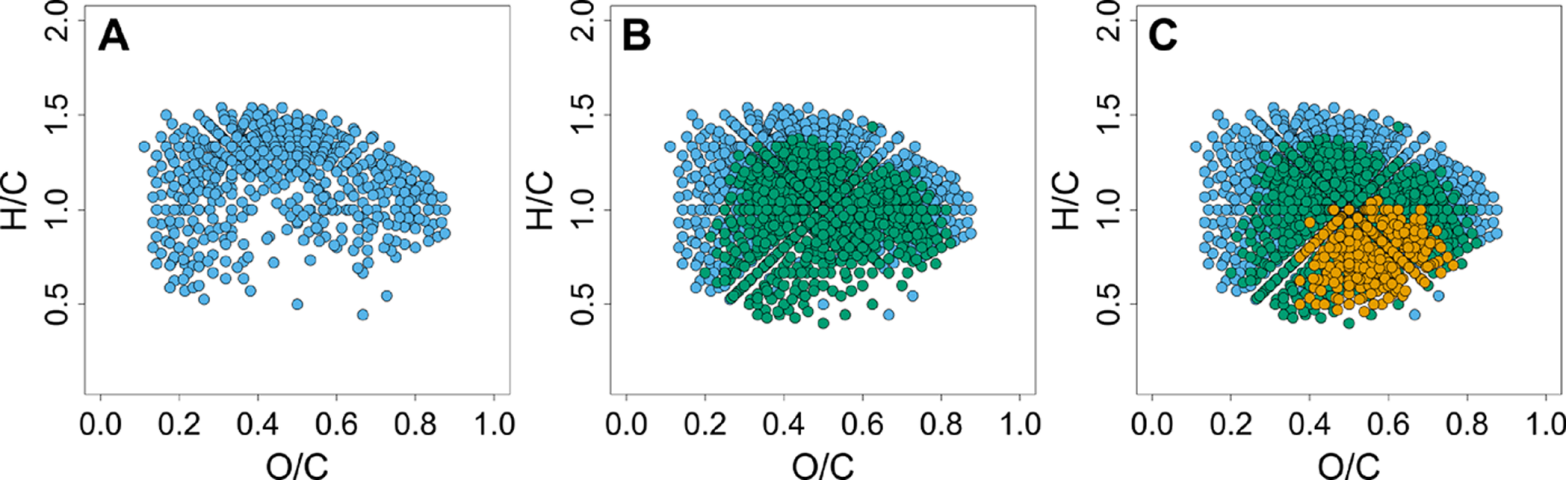
Van Krevelen diagrams of the control (treatment 1, second technical replicate). The colors indicate the number of identified ketone functionalities for the respective molecular formulas: A) Molecular formulas without identified ketone functionality (549 formulas). B) Molecular formulas without (blue) and with one (green, 740 formulas) identified ketone functionality. C) Molecular formulas without (blue), with one (green) and with two (orange, 297 formulas) identified ketone functionalities. Ketones were identified based on the comparison with the sample treated with unlabeled NH_4_OAc (treatment 3, second technical replicate).

To investigate the molecular properties of ion masses representing compounds containing keto groups, we assigned molecular formulas to these masses. Note that molecular formula assignment is not possible for all detected masses.^32^ The amination affected molecular formulas essentially over the entire H/C and O/C range of 0.5–1.4 and 0.2–0.8, respectively (Figure 3). Thus, compounds with ketone functionalities are widely distributed over the van Krevelen space. Compounds with these H/C and O/C values are frequently assigned as lignin- (0.1 < O/C ≤ 0.6) or tannin-derived (O/C > 0.6).^37^ Lignin is composed of phenylpropane units and tannins most often consist of either flavonoid (condensed tannins) or gallic acid (hydrolyzable tannins) derivatives. Therefore, tannins likely exhibit a more pronounced aromatic character than lignin and contain plenty ketone functionalities. Possibly, most of the keto-rich components (orange dots in Figure 3) are associated with a tannin origin.

In addition, a clear trend was observed in the van Krevelen space: The number of ketones per molecular formula increased as the O/C values increased, and the H/C values decreased (which correlates with the double bond equivalents). No ketone containing species were detected below O/C values of 0.2, and the presence of two ketone functionalities was essentially limited to O/C values above 0.4 and H/C values below 1.0. This can simply be explained on a structural level since the presence of two ketones requires the presence of more oxygen and more double bond equivalents.

**Figure 4:**
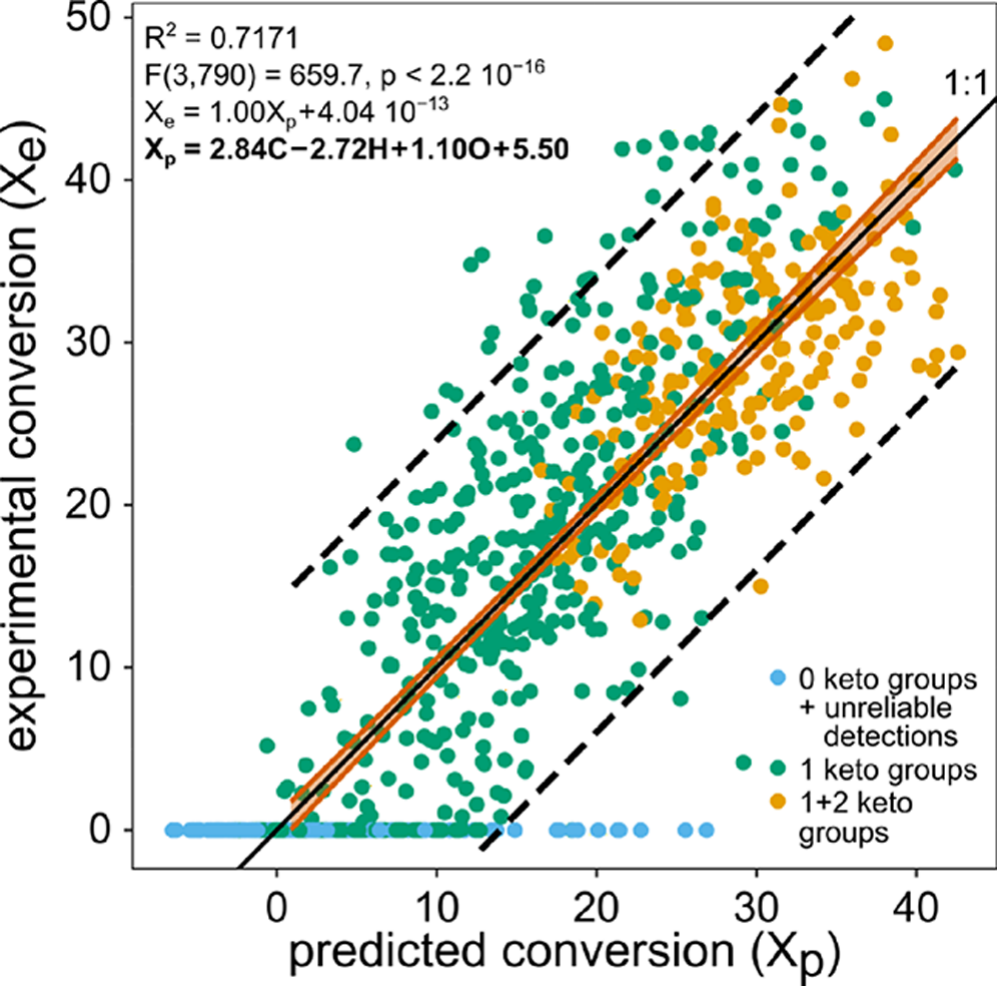
Experimental conversion of the reductive amination (X_e_) as a function of the predicted conversion (X_p_). Each dot represents the conversion of a given molecular formula. Blue: molecular formulas lacking or uncertain to contain ketone moieties, green: molecular formulas with exactly one, and orange: molecular formulas with one and two detected ketone moieties. The experimental conversion was calculated from the educt mass peaks of the control (treatment 1, second technical replicate) and treatment 3 (second technical replicate) after molecular formula attribution. Prior to multiple linear regression, intensity values less than 20% of the maximum detected intensity for the control were removed and negative conversions as well as conversions associated with molecular formulas unreliably or not identified to contain ketone moieties were set to zero. Confidence (ocher line) and prediction bands (dashed black line) were calculated for a significance level of *p* = 0.05. The identity (1:1) line (solid black line) lies within the confidence bands of the linear regression.

This trend along the O/C and H/C axes becomes even more evident when investigating the conversions as a function of the O/H values (Figure S14). Conversion rates of individual molecular formulas significantly correlated with the respective O/H values. Such correlation is highly surprising. For single compounds either a conversion of 100% or 0% is expected, depending on whether or not they bear ketone moieties as structural features and assuming quantitative conversion. Consequently, no correlation between the conversion and the O/H values is expected for single compounds. For example, in the case of isomers with the molecular formula C_9_H_8_O_4_ (O/H = 0.50), 100% conversion is anticipated for ketone-containing compounds such as 4-hydroxyphenylpyruvic acid and 0% conversion is expected for any dihydroxycinnamic acid with the same molecular formula and O/H value due to lacking ketone moieties. In contrast, the conversion of SRNOM compounds significantly correlated with the O/H values (Figure S14). The predictability of the conversion can be further improved when applying a multiple linear regression model based on the number of C, H and O of attributed molecular formulas (Figure 4). Thus, the proportion of ketone-containing isomers represented by a given molecular formula is predictable solely based on its elemental composition. This indicates a stochastic occurrence of ketone moieties across SRNOM compounds in line with the central limit theorem. A similar observation was made regarding the occurrence of carboxyl functionalities in deep-sea DOM.^4^

We also investigated the distribution of molecular formulas that reliably represented ketone-containing isomers across the different compound classes putatively assigned to molecular formulas (Supporting Information, 2.8). Overall, molecular formulas containing ketone isomers occurred representatively across the compound classes. However, molecular formulas containing exactly one keto group were relatively more abundant in the highly unsaturated fraction. In contrast, aromatic compounds contained an overproportionally high number of molecular formulas containing one and two keto groups. This is consistent with the observation by Leenheer et al.^38^ that ketones in Suwannee River fulvic and humic acids predominantly occur in aromatic structures, with a frequency of about one ketone per monocyclic aromatic ring.

With all these results in hand we estimated the amount of oxygen in SRNOM that is bound in the form of ketone moieties. Our conservative estimates are that about 28% of the molecules in SRNOM contain a keto group. Considering in addition our estimates on the number of keto groups per molecular formula, 22.7% of the molecules contained at least one, 5.6% contained at least two and 0.2% contained three ketones. The intensity-weighted mean oxygen content for the respective control was 9.8. This implies that at least:
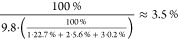
of the oxygen in SRNOM is bound in the form of ketones. This value represents the minimum content of oxygen that is bound in the form of ketones detectable by mass spectrometry, as it is likely that not all ketones were converted under the applied reaction conditions, most likely due to steric or electronic effects. In addition, not all reaction products may have been identified, e.g., due to the applied detection ratio. The upper limit of oxygen bound in the form of ketones in SRNOM was calculated based on elemental analysis^30^ and ^13^C NMR spectroscopic^39^ data derived from the IHSS and was 13.1% (for the calculation we refer to S2.9, Supporting Information). Thus, we can constrain the proportion of oxygen that is bound in ketone functionalities in SRNOM between 3.5 and 13.1%.

### 3.4 Reductive Amination Using Isotopically Labeled ^15^NH_4_OAc

Although reaction times of 168 h led to the detection of increased numbers of molecular ions containing ketone functionalities, we performed the isotope labeling experiment with a reaction time of 48 h because of the significant loss of SPE-DOC at longer reaction times (Table 1). The reactions were performed with and without molecular sieve applying optimized reaction conditions (10 equiv NH_4_OAc and 2 equiv NaBH_3_CN, Table 2). As for the unlabeled samples, SPE-DOC decreased due to the reductive amination and when using molecular sieve, while SPE-TDN as an indicator for the incorporation of nitrogen increased (Table 2).

**Table 2:** Reaction Conditions for the Reductive Amination of SRNOM with Isotopically Labeled ^15^NH_4_OAc^e^

#	^15^NH_4_OAc (equiv)	NaBH_3_CN (equiv)	MS^a^ (3 Å)	time (min)	SPE-DOC (μM)	SPE-TDN (μM)	EE^b^	reliably detected reaction products^c^
11	0	0	n	48 h	64.52	1.23	84	0–7
12	10	2	n	48 h	50.15	2.20	65	749–837
13	0	0	y^d^	48 h	48.05	1.65	53	3–7
14	10	2	y^d^	48 h	46.09	1.91	50	281–467

a MS: molecular sieve.

b EE: extraction efficiency based on DOC, calculated with respect to a control containing 66.65 μmol prior to extraction. This control was prepared by dissolving 2.02 mg SRNOM in anhydrous MeOH (2 mL), evaporating the MeOH under a stream of nitrogen at 50 °C and redissolving the residual in 250 mL ultrapure water acidified with hydrochloric acid (∼8 mol/L) to pH 2.

c The range is given for all possible sample-control combinations. For treatments 11 and 13, the number of reliably detected reaction products indicates the false positive detections.

d Approximately 80 mg of molecular sieve was used.

e Equivalents (equiv) are based on the total oxygen content of SRNOM. All reactions were performed in triplicates.

The incorporation of ^15^N has the advantage that reaction products are more readily detectable based on the specific mass difference of 2.0287 Da per ketone moiety that was converted to an amino group because ^15^N occurs naturally at only 0.37%. As such, no threshold in the form of a detection ratio may be needed when using ^15^NH_4_OAc as the nitrogen source. The isotopic label is also of advantage to differentiate product peaks that are not clearly assignable to a single educt (*cf*. 3.3). As expected, the false positive rate was low (∼4%) when no detection ratio was applied (i.e., *dr* = 1.0). In contrast, a detection ratio of 1.0 yielded false positive rates exceeding 20% for some treatments using unlabeled NH_4_OAc. However, to obtain false positive rates <1%, we had to apply detection ratios up to 1.5 for the isotopically labeled sample set (*cf*. Supporting Information, S2.6).

As expected, a few reaction products (up to 136) with mass differences of n*1.0316 Da (*n* = 1, 2 or 3) were observed in the experiments using ^15^NH_4_OAc. According to the manufacturer, the reagent was labeled to 98% and the few unlabeled reaction products are likely due to traces (2%) of ^14^N in the reagent. If this is true, these detections should predominantly occur for mass peaks with high intensities. To support this hypothesis, we exemplarily analyzed selected samples and found that more than 85% of these detections were observed for the 10% of most abundant peaks. The low abundance of unlabeled products furthermore confirms our statistical approach for identifying false positives. Considering mass differences of n*2.0287 Da, which are indicative for the presence of isotopically labeled reaction products (Figure 1C), we identified up to 837 molecular ions representing isomers with ketones. This number decreased to only up to 467 molecular ions when molecular sieve was used during the reaction. Thus, less ketones were detected when using ^15^NH_4_OAc for the reaction compared to the ^14^N counterpart. In principle, the observed differences between the ^15^N-labeled and unlabeled approaches could be because of potential overestimation of the number of ketone groups in the unlabeled approach. However, we chose a conservative and statistically sound approach for the identification of reaction products through comparison with control samples, which makes overestimation unlikely. Kinetic isotope effects of heavy atoms such as ^14^N/^15^N are usually small and strong isotope effects on the ionization selectivity are unlikely. A likely explanation for this finding is that ^15^N-labeled products do not naturally occur, and they are only detectable if their abundance exceeds the detection threshold. In contrast, product peaks when using unlabeled NH_4_OAc may already be present prior to the derivatization and potential intensity changes are thus more readily detected (S2.5, Supporting Information).

### 3.5 2D NMR Spectroscopic Evidence for the Amination of Ketone Moieties in SRNOM

**Figure 5:**
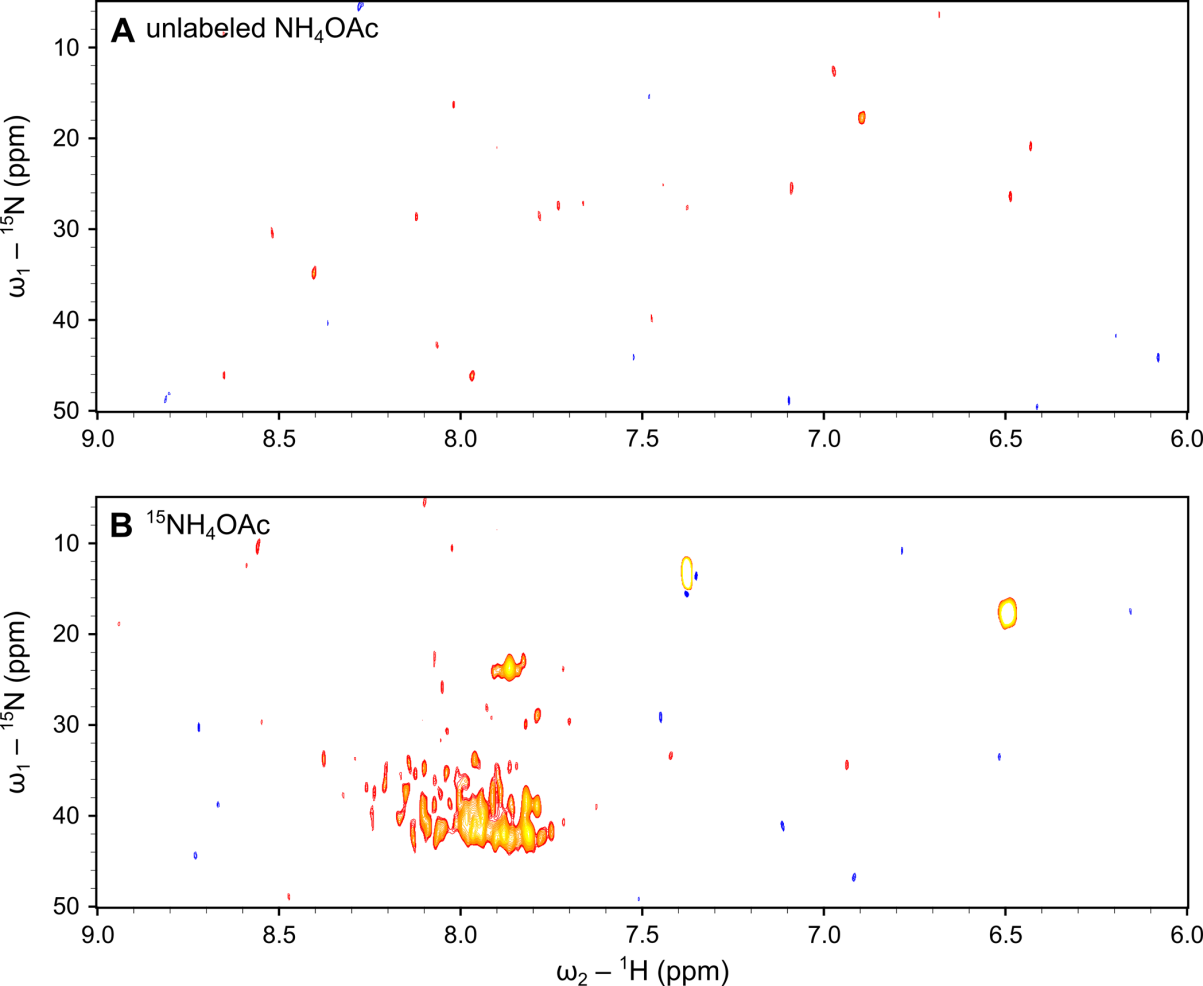
2D NMR spectroscopic evidence for the reductive amination of SRNOM. Comparison of 2D ^1^H,^15^N heteronuclear single quantum coherence (HSQC) spectra of SRNOM treated with A) unlabeled NH_4_OAc (treatment 3, second technical replicate) and B) ^15^NH_4_OAc (treatment 12, second technical replicate). No signals being representative for reaction products were detected in the HSQC spectrum of SRNOM treated with unlabeled NH_4_OAc due to the low natural isotopic abundance of ^15^N (0.37%). In contrast, the HSQC spectrum of SRNOM treated with ^15^NH_4_OAc exhibited numerous nitrogen-proton one-bond correlations, as the ^15^N-enrichment in reaction products (∼100%) drastically improved the NMR detection sensitivity by more than 2 orders of magnitude. The NMR spectra were recorded at 298 K in CD_3_OD using a Bruker Avance Neo 800 MHz (for ^1^H) instrument equipped with a 5 mm BBO cryoprobe. Experimental time was 39 h.

We recorded 2D ^1^H,^15^N heteronuclear single quantum coherence (HSQC) and 2D ^1^H,^15^N heteronuclear multiple bond correlation (HMBC) NMR spectra, displaying one-bond correlations and long-range (most often ^2^*J* and ^3^*J*, occasionally ^4^*J*) correlations, respectively. As expected from the poor sensitivity of ^15^N NMR at natural isotopic abundance, SRNOM treated with unlabeled NH_4_OAc showed no signals in the HSQC spectrum (Figure 5A). In contrast, SRNOM treated with ^15^NH_4_OAc exhibited numerous correlations in the HSQC spectrum (Figure 5B), highlighting the successful amination of keto groups in distinct molecular structures of SRNOM. The observed chemical shifts of nitrogen and proton in the range of 20–45 ppm and 7.5–8.5 ppm, respectively, indicate the formation of primary amines (*cf*. discussion in Supporting Information, S2.10), which were present in protonated form due to the acidification of samples to pH 2 prior to SPE. The large chemical shift dispersion of approximately 25 ppm in the nitrogen and 1 ppm in the proton dimensions of HSQC, highlights that ketone moieties occur in highly diverse chemical environments in SRNOM compounds. The implementation of nonuniform sampling in ^1^H,^15^N HSQC (50% sampling rate, Figure S17, Supporting Information) allowed us almost to triple the number of scans to 1400 within a reasonable time frame (53 h). The increase in scan numbers led to an increase of detected peaks from 50 (uniformly sampled) to 88 (nonuniformly sampled), thus facilitating the detection of new signals that would otherwise be buried in the noise level or overlap with other signals due to lower resolution. The long-range proton-nitrogen correlations appeared in the HMBC spectrum (Supporting Information, Figure S18) of SRNOM treated with ^15^NH_4_OAc further supported the reductive amination of ketone functionalities in SRNOM. However, here we observed much less signals.

Therefore, at least a subset of amines likely resides in similar chemical environments, producing detectable HMBC signals, while other amines may disperse across diverse chemical environments, resulting in signals too weak for detection above background noise. This scenario implies that a significant portion of ketones in SRNOM is primarily bound in structural motifs with quaternary carbons three bonds away in one residue, and similar motifs on the second residue. This aligns with Leenheer et al.^38^ findings that most ketones in Suwannee River fulvic acid exist as aryl-alkyl and diaryl ketones. In aryl-alkyl ketones with similar alkyl substituents within the first connection spheres and different substitutions on the aryl residue (such as electron-withdrawing and electron-donating groups in different substitution pattern), only a few HMBC-correlations but various HSQC-correlations would be expected.

### 3.6 Environmental Implications and Practical Advice

Here, we showed that simple and mild reaction conditions lead to the conversion of ketone moieties present in DOM to primary amines. The number of molecular formulas containing nitrogen (intensity-weighted) increased by more than 20-fold due to the derivatization. Bulk TDN concentrations increased by up to 275%. In addition, the reductive amination selectively yielded nitrogen containing compounds, since 86–97% of the reaction products contained nitrogen. We estimated that at least 3.5% but not more than 13.1% of the oxygen in SRNOM is bound in keto groups. Whereas the proportion of peaks detected as amination products only slightly increased with longer reaction times, conversion rates and mole percentages of ketone-containing species were significantly higher when turning to longer reaction times. The drawback of longer reaction times was a decrease in SPE recoveries. Although we showed that using ^15^NH_4_OAc for the reductive amination facilitates the identification of reaction products (i.e., lower detection ratios were needed), we demonstrated that unlabeled NH_4_OAc can be used at least as well to analyze the sample with respects to its ketone-containing compounds. Besides the demonstrated mass spectrometric applications, the present study offers a new and simple synthetic strategy for the targeted ^15^N NMR spectroscopic analysis of ketone-containing compounds in DOM. We exemplarily demonstrated this by recording ^1^H,^15^N HSQC and HMBC NMR spectra of sample amounts corresponding to less than 20 μg of nitrogen and showed that exclusively primary amines were formed as reaction products. When using larger sample amounts, other NMR spectroscopic experiments can be conducted to obtain information on specific structural motifs in which ketones occur in SRNOM. Our study paves the way and provides novel methods for follow-up studies that focus on comparing samples across diverse aquatic environments.

## Supporting Information Available

Supplementary methods (S1): Reductive amination of individual compounds (S1.1); Melonoside A as an example for the functional group selectivity of the reductive amination (S1.2); FT-ICR-MS measurements (S1.3); NMR acquisition and processing parameters (S1.4); Identification of molecular ions containing ketones based on the Kendrick mass defect (S1.5); Detection ratio (S1.6); Scenarios yielding to the detection of ketone-containing molecular species (S1.7); Selected scenarios leading to potential double counting of peaks identified as products (S1.8); Conversion (S1.9); Estimation of the molar proportion of ketone-containing compounds (S1.10). Supplementary results (S2): ^1^H NMR spectra of isolated products (S2.1); Principal coordinate analysis (PCoA) of the isotopically unlabeled samples (S2.2); Selected sections of exemplary mass spectra (S2.3); Exemplary mass spectra demonstrating the detection of reaction products (S2.4); Exemplary mass spectra demonstrating the facilitated detection of an increase in peak intensity compared to the detection of a new peak (S2.5); Detected ketones (S2.6); Experimental conversion as a function of O/H values (S2.7); Compound group classification of molecular formulas (S2.8); Calculation of the upper oxygen content that is bound in the form of ketone moieties in SRNOM (S2.9); Identification of primary amines as reaction products by NMR (S2.10); NMR spectra of SRNOM (S2.11); References (PDF)
