# Supplementary material for: Dissolved Organic Matter Contains Ketones Across a Wide Range of Molecular Formulas
Source: Environ Sci Technol. 2024 Aug 20;58(35):15587–97. doi: 10.1021/acs.est.4c02593 (PMC11375772; doi:10.1021/acs.est.4c02593)
Supplement: Supplementary file 1 — es4c02593_si_001.pdf [file es4c02593_si_001.pdf]

Supporting Information

Dissolved Organic Matter Contains Ketones Across a Wide Range of Molecular Formulas

Nico Mitschke[1,*](0000-0002-1043-7199), Sahithya Phani Babu Vemulapalli[1](0000-0001-7773-7527), Thorsten Dittmar[1,2](0000-0002-3462-0107)

[1]Institute for Chemistry and Biology of the Marine Environment (ICBM), School of Mathematics and Science, Carl von Ossietzky Universität Oldenburg, Ammerländer Heerstraße 114–118, 26129 Oldenburg, Germany.

[2]Helmholtz Institute for Functional Marine Biodiversity (HIFMB) at the Carl von Ossietzky Universität Oldenburg, Ammerländer Heerstraße 231, 26129 Oldenburg, Germany.

https://uol.de/en/icbm/marine-geochemistry

E-mail: nico.mitschke@uni-oldenburg.de

sahithya.phani.babu.vemulapalli@uni-oldenburg.de

thorsten.dittmar@uni-oldenburg.de

*author to whom correspondence should be addressed

Contents: 19 Figures, 11 Tables, 1 Scheme, References

This document contains supporting information for the above-mentioned manuscript and consists of 41 pages with supplementary methods, supplementary results, and references.

Table of Contents

S1 Supplementary methods 3

S1.1 Reductive amination of individual compounds 3

S1.2 Melonoside A as an example for the functional group selectivity of the reductive amination 5

S1.3 FT-ICR-MS measurements 6

S1.4 NMR acquisition and processing parameters 8

S1.5 Identification of molecular ions containing ketones based on the Kendrick mass defect 9

S1.6 Detection ratio 10

S1.7 Scenarios yielding to the detection of ketone-containing molecular species 12

S1.8 Selected scenarios leading to potential double counting of peaks identified as products 14

S1.9 Conversion 15

S1.10 Estimation of the molar proportion of ketone-containing compounds 16

S2 Supplementary results 17

S2.1 ^{1}H NMR spectra of isolated products 17

S2.2 Principal coordinate analysis (PCoA) of the isotopically unlabeled samples 19

S2.3 Selected sections of exemplary mass spectra 20

S2.4 Exemplary mass spectra demonstrating the detection of reaction products 24

S2.5 Exemplary mass spectra demonstrating the facilitated detection of an increase in peak intensity compared to the detection of a new peak 27

S2.6 Detected ketones 28

S2.7 Experimental conversion as a function of O/H values 31

S2.8 Compound group classification of molecular formulas 32

S2.9 Calculation of the upper oxygen content that is bound in the form of ketone moieties in SRNOM 33

S2.10 Identification of primary amines as reaction products by NMR 34

S2.11 NMR spectra of SRNOM 35

References 39

S1 Supplementary methods

S1.1 Reductive amination of individual compounds

General procedure for the reductive amination with single substrates

A solution of NH_4OAc (10 equiv.) in methanol (5 mL/mmol) was added to the individual ketones (1 or 2 mmol) in 20 mL amber glass vials capped with a septum and the mixture was stirred for 30 min. After a period of 30 min, $NaBH_3CN$ (2 equiv.) was added in one portion. After stirring for 48 h at ambient temperature, the reaction mixture was acidified with hydrochloric acid (12 mol/L, approx. 2 mL/mmol) to pH 2 and water (25 mL) was added. The aqueous layer was washed with dichloromethane (3 x 20 mL), basified with aqueous KOH (5 mol/L, approx. 4 mL) to pH 10 and extracted with dichloromethane (4 x 20 mL). The combined organic layers were dried over $MgSO_4$, filtered, and evaporated.

We monitored the reactions with GC-MS and complete conversion was observed for all tested substrates after 48 h. We decided to not isolate all products because most of the transformations have already been reported to take place with >80% yield under almost identical or very similar reaction conditions (*cf.* Table S1). Since the transformation of 1-tetralone using NH_4OAc and $NaBH_3CN$ has yet not been reported and the transformation of 4-phenyl-2-butanone was only reported with yields of 43–52%[1], we decided to isolate the respective products, 1-phenylethylamine and 1-methyl-3-phenylpropylamine, and found yields of 83 and 98%, respectively.

GC-MS detection of individual compounds

GC-MS analysis was performed on a GC-MS-QP2020 (Shimadzu Corp.) with an OPTIMA 5 HT fused silica capillary column (Macherey-Nagel, 30 m length, 0.25 mm internal diameter, 0.25 µm film thickness) and helium as carrier gas. The GC oven temperature was programmed from 50 °C (3 min hold time) to 280 °C at a rate of 40 K min^{-1} (8.5 min hold time). The MS was operated in EI mode (70 eV) at a source temperature of 230 °C and a transfer line temperature of 280 °C. The mass range was 50–600 Dalton at a scan cycle time of 0.2 s.

NMR spectroscopy of individual compounds

^1H NMR spectra were acquired on an Avance DRX 300 instrument (Bruker BioSpin). Chemical shifts were reported in parts per million (ppm) and coupling constants were calculated in Hertz (Hz). Deuterated chloroform ($CDCl_3$) was used as solvent and the signal of the residual protons of the solvent was used for calibrating the spectra. NMR spectra of Suwannee River natural organic matter (SRNOM) derived samples were acquired on a high-field Bruker Avance Neo 800 instrument (Bruker BioSpin) as detailed in the main manuscript and in S1.4.

Table S1 Individual compounds that were tested in reductive aminations. Complete conversion was observed for all substrates by monitoring the reactions with GC-MS.

Educt	Product	Yield (literature)
5-Nonanone	5-Aminononane	86%[2]
Acetophenone	1-Phenylethylamine	77%[3]
1-Tetralone	1,2,3,4-Tetrahydro-1-naphthylamine	83%[+]
Dicyclohexyl ketone	*N*,*N*-Dicyclohexylmethylamine	63%[4]
4-Phenyl-2-butanone	1-Methyl-3-phenylpropylamine[1]	43–52%[1*], 98%[+]
Cyclohexyl phenyl ketone	Cyclohexyl(phenyl)methanamine	92%[5]

[+]isolated yield after liquid-liquid extraction.
[*]only a range for the yield of several products, including 4-phenyl-2-butanone, was provided in the reference.

S1.2 *Melonoside A as an example for the functional group selectivity of the reductive amination*

Reductive aminations applying the reaction conditions used in this study are highly selective with respect to the transformation of ketones and aldehydes. As an example for the functional group selectivity, we refer to the reductive amination of melonoside A, a complex fatty acid amide, which was selectively converted under almost identical reaction conditions as applied in this study to the corresponding diamine (Scheme 1).[6]

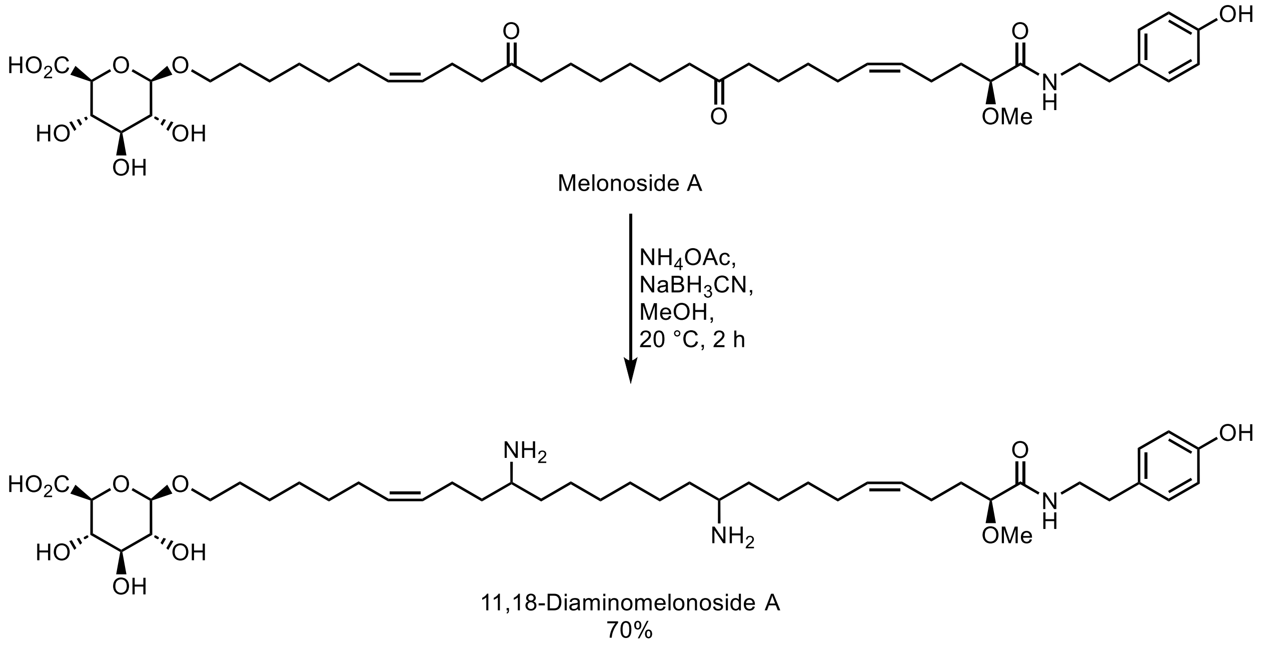

Scheme 1 Conversion of melonoside A to the corresponding diamine (11,18-diaminomelonoside A) by reductive amination applying identical reaction conditions (except for the reaction time) as used in this study.[6]

S1.3 FT-ICR-MS measurements

Methanol extracts from SPE were diluted by approx. factor 24 with methanol and ultrapure water to a final ratio of methanol/water 1:1 (*v*/*v*), filtered through pre-rinsed 0.2 µm PTFE syringe filters and analyzed in a random order by injection with a PAL 3 autosampler (CTC Analytics). The ESI capillary voltage was set to 4.5 kV. Spectra were acquired in duplicate measurements with a quadrupole accumulation time of 0.2 s over a scan-range from *m*/*z* 91 to *m*/*z* 2000 with 200 accumulated scans per sample and were internally calibrated with the DataAnalysis software (Bruker Daltonics) using a mass list containing 74 compounds with *m*/*z* values ranging from *m*/*z* 225.0405 to *m*/*z* 621.2553. A mass error <0.1 ppm was achieved after calibration. The spectra were exported as .csv data files using in-house written scripts and further processed with ICBM-OCEAN[7] using default parameters, if not otherwise noted below. For samples from treatments using unlabeled NH_4OAc, peaks with intensities greater than three times the method detection limit (MDL) were assigned to molecular formulas within the range $^{12}C_{1-65}{}^{1}H_{1-180}{}^{16}O_{1-50}{}^{14}N_{0-7}{}^{32}S_{0-2}{}^{31}P_{0-1}$ and without using the NSP rule (see Merder *et al*.[7] for further explanations). The homologous series network filter was set to CH_2, CO_2, H_2 and H_2O, and no molecular formulas with intensities present below chosen percentage of samples were excluded. For further analysis, only the likeliest matches dataset was used. After formula attribution, all molecular formulas containing ^{13}C, ^{18}O, ^{15}N, ^{34}S or ^{37}Cl as well as those with m/z ≥ 800, H/C ≥ 2.5 and O/C ≥ 1 were removed. Peaks with intensities that visually deviated significantly from the overall log-normal distribution of the mass spectrum were considered as contaminants and were removed. To compensate fluctuating measurement conditions (i.e., sample concentrations and analytical reproducibility), the MDL of all samples was adjusted to the MDL of the sample with the lowest absolute intensity according to:

$$\mathrm{MDL_{adj}} = \frac{\mathrm{I_{sample}}}{\mathrm{I_{min}}} \cdot \mathrm{MDL}$$

with:

$\mathrm{MDL_{adj}}$: adjusted method detection limit for a chosen peak of the sample.

$\mathrm{I_{sample}}$: total spectral intensity of the sample.

$\mathrm{I_{min}}$: minimal total spectral intensity of all samples.

MDL: method detection limit for a chosen peak of the sample.

Peaks below the adjusted MDL were deleted.

For parameters displayed in Table 1 of the main manuscript, analytical and technical replicates were averaged with the restriction that a detected peak was only considered for further analysis if detected in two of four (treatments with 48 h reaction time) or three of six (treatments with 168 h reaction time) replicates, regardless of the replicate type. Averages were calculated only for peaks with intensities unequal to zero. For the analysis of individual technical replicates,

analytical replicates were averaged if a molecular formula was detected in both replicates. The sum of intensities of all remaining peaks was sample wise normalized to 10,000.

The identification of ketone-containing species was performed with an in-house written MATLAB routine that worked on the junction files (i.e., peak lists without molecular formula assignments) of ICBM-OCEAN. For this purpose, analytical replicates were averaged if a peak was detected in both replicates and molecular ion masses with $m/z > 800$ were deleted. Peaks that were identified as contaminants were removed as described above. No MDL correction was used in this case because we wanted to study the maximal detectable number of molecular formulas representing isomers with ketone moieties.

S1.4 NMR acquisition and processing parameters

Table S2 2D NMR acquisition parameters.

Experiment	Regular $^1H,^{15}N$ HSQC		50% NUS $^1H,^{15}N$ HSQC		$^1H,^{15}N$ HMBC	
Pulse sequence	hsqcedetgpsisp2.2		hsqcedetgpsisp2.2		hmbcgpndqf	
Dimensions	F2	F1	F2	F1	F2	F1
TD	2048	128	2048	128	2048	128
SWH (Hz)	9615	10000	9615	4065	9615	10000
DS	32		32		32	
NS	512		1400		512	
D1 (sec)	2		2		2	
$^1J_{N\text{-}H}$ (Hz)	90		90		-	
$^nJ_{N\text{-}H}$ (Hz)	-		-		10	
Trim pulse (µsec)	1000		1000		-	
CPD/BB decoupling	garp4		garp4		no decoupling	
180° shaped pulse for inversion	Crp60,0.5,20.1, 500 µsec		Crp60,0.5,20.1, 500 µsec		-	
180° shaped pulse for refocusing	Crp60comp.4, 2000 µsec		Crp60comp.4, 2000 µsec		-	
NUS schedule*	-		sinusoidal-weighted Poisson-gap[8,9]		-	
Experiment time	1 day 15 hours		2 days 5 hours		1 day 15 hours	

*sinusoidal-weighted Poisson-gap sampling schedules were generated using the Schedule Generator Version 3.0 available on nus@HMS webpage.[10]

Table S3 2D NMR processing parameters.

Experiment	Regular $^1H,^{15}N$ HSQC		50% NUS $^1H,^{15}N$ HSQC		$^1H,^{15}N$ HMBC	
Dimensions	F2	F1	F2	F1	F2	F1
SI	4096	2048	4096	2048	4096	2048
WDW	QSINE; SSB = 2	QSINE; SSB = 2	QSINE; SSB = 2	QSINE; SSB = 2	QSINE; SSB = 0	QSINE; SSB = 0
PH_mod	pk	pk	pk	pk	no	mc
NUS reconstruction	-		CS-IST[11,12]		-	

S1.5 Identification of molecular ions containing ketones based on the Kendrick mass defect

In addition to the MATLAB routine described in the main manuscript, we also followed an approach relying on the Kendrick mass defect (KMD). For this approach, the Kendrick masses (KM) specific for the reductive amination of a ketone (loss of oxygen, formal incorporation of NH_3) were calculated for all molecular formulas as follows:

$$KM = \frac{m_{nominal}(NH_3) - m_{nominal}(O)}{m_{exact}(NH_3) - m_{exact}(O)} \cdot M = \frac{17 - 16}{17.026549 - 15.994915} \cdot M = 0.969336 \cdot M$$

with:

M: observed mass of peaks with attributed molecular formulas.

From the KM, the KMD was calculated as follows:

$$KMD = M_{nominal} - KM$$

with:

$M_{nominal}$: nominal mass of peaks with attributed molecular formulas.

In principle, the attribution of molecular formulas is not absolutely necessary in our case since the observed *m*/*z* values can be used for the calculation of the Kendrick mass and can be rounded to derive the nominal mass since expected mass defects in the analyzed mass range are much smaller than 0.5 Da. Molecular formulas/molecular ions with identical KMD (for measured data a tolerance for comparing KMDs must be used) are part of a network of ketone containing species and their amination products. This approach has the advantage that it is already easy implemented in a spreadsheet editor such as Microsoft Excel and is also helpful for mass detectors with low mass accuracy in the higher mass range. We observed similar results for both approaches but herein only results for the MATLAB routine are reported.

S1.6 Detection ratio

As a prerequisite for the detection of reaction products, we defined that the mass peak of a potential educt was detected in the control samples. However, it is very likely that a peak that can be attributed to a nitrogen containing compound, thus potentially representing a product peak, was present in the control sample but was also (partially) caused due to nitrogen containing compounds formed by the reductive amination. This circumstance is schematically depicted in Figure S1. Thus, it is not sufficient to only attribute peaks to as products of the reductive amination, which were not detected in the control sample.

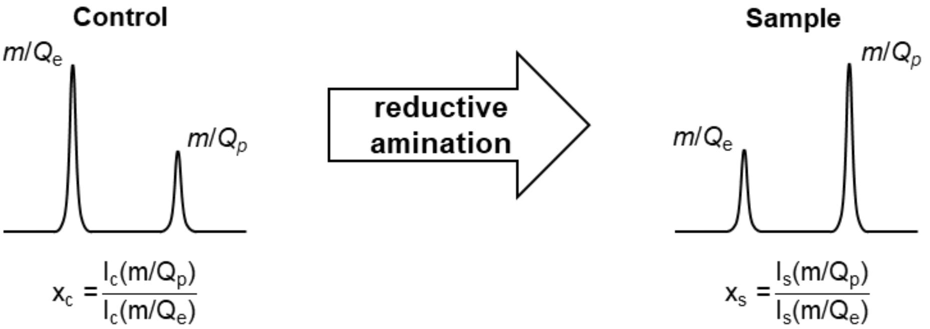

Figure S1 Schematic representation of changing ratios of product and educt peak intensities due to reductive amination.

As depicted in Figure S1, reductive amination processes are among other possibilities detected if the intensity ratio of product and educt in the derivatized sample (x_s) is larger than in the control (x_c): $x_s > x_c$. Since the ratio between the intensities of product and educt peaks is likely to vary especially among technical replicates, we defined a constant referred to as detection ratio *dr*, representing a threshold that the ratio x_s must exceed that of x_c to indicate a positive detection:

$$\frac{I_s(m/Q_p)}{I_s(m/Q_e)} > dr \cdot \frac{I_c(m/Q_p)}{I_c(m/Q_e)}$$

with:

$I_s(m/Q_p)$: molecular ion signal intensity of the putative reaction product in the sample.
$I_s(m/Q_e)$: molecular ion signal intensity of the putative reaction educt in the sample.
$I_c(m/Q_p)$: molecular ion signal intensity of the putative reaction product in the control.
$I_c(m/Q_e)$: molecular ion signal intensity of the putative reaction educt in the control.
dr: detection ratio that must be exceeded to indicate a positive detection.

We considered potential fluctuations in sample concentrations and measurement sensitivity, which yield to several cases that indicate reliable or unreliable detections (S1.7). If not otherwise noted, we only considered reliable detections for further analysis.

The detection ratio was individually determined for the three different experimental sets (unlabeled, 48 h reaction time; unlabeled, 168 h reaction time; ^{15}N-labeled, 48 h reaction time) by screening 33 detection ratios ranging from 1.00 to 10.0 (1.00, 1.10, 1.20, 1.30, 1.40, 1.50, 1.60, 1.70, 1.80, 1.90, 2.00, 2.25, 2.50, 2.75, 3.00, 3.25, 3.50, 3.75, 4.00, 4.25, 4.50, 4.75, 5.00, 5.50, 6.00, 6.50, 7.00, 7.50, 8.00, 8.50, 9.00, 9.50, 10.0) and choosing the lowest detection ratio that yielded less than 1% of false positives for all treatments of a sample set using a specific control (*cf*. S2.6). For this, molecular ions that were also detected as reaction products among controls were considered as false positives. In Figure S2 we show exemplarily the number of reliably detected ketone-containing species and their mean conversion as a function of the detection ratio for treatment 3 (second technical replicate) using treatment 1 (second technical replicate) as control. Whereas the number of reliable detections continuously decreased with increasing detection ratio, the conversion initially increased up to a detection ratio of 2.5 to then decrease again. For this combination of derivatized sample and control, the false positive rate was less than 0.1% for the optimized detection ratio of 1.6 (dashed line). Using this detection ratio, 1209 molecular ions were reliably attributed to represent at least one species with at least one keto group and the mean conversion of these 1209 molecular ions was 29.2%. Apart from the controls, all derivatized samples showed a similar pattern of reliable detections and conversions in dependence of the detection ratio.

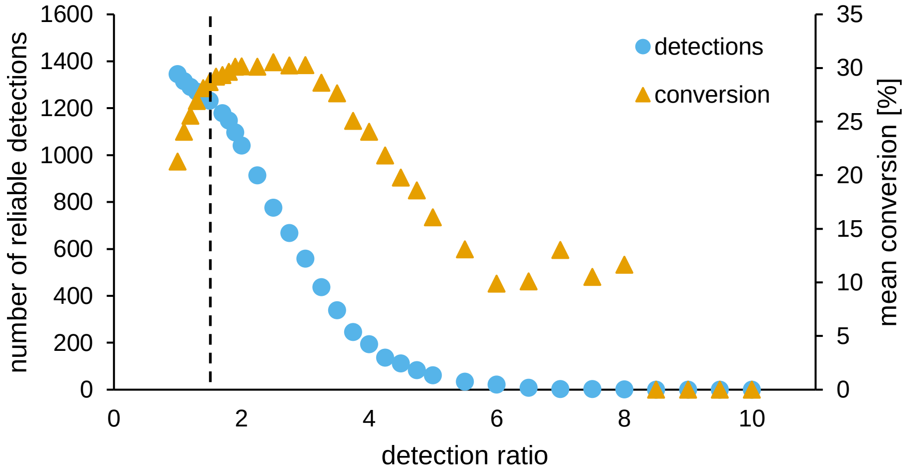

Figure S2 Number of reliable detections (blue) and conversion (orange) as a function of the detection ratio *dr* for treatment 3 (second technical replicate) using treatment 1 (second technical replicate) as control. The dashed line indicates the optimized detection ratio of 1.6 for this setup.

S1.7 Scenarios yielding to the detection of ketone-containing molecular species

To compensate varying analytical sensitivities and varying concentrations among samples, several cases leading to the reliable detection of reaction products were considered as depicted in Figure S3.

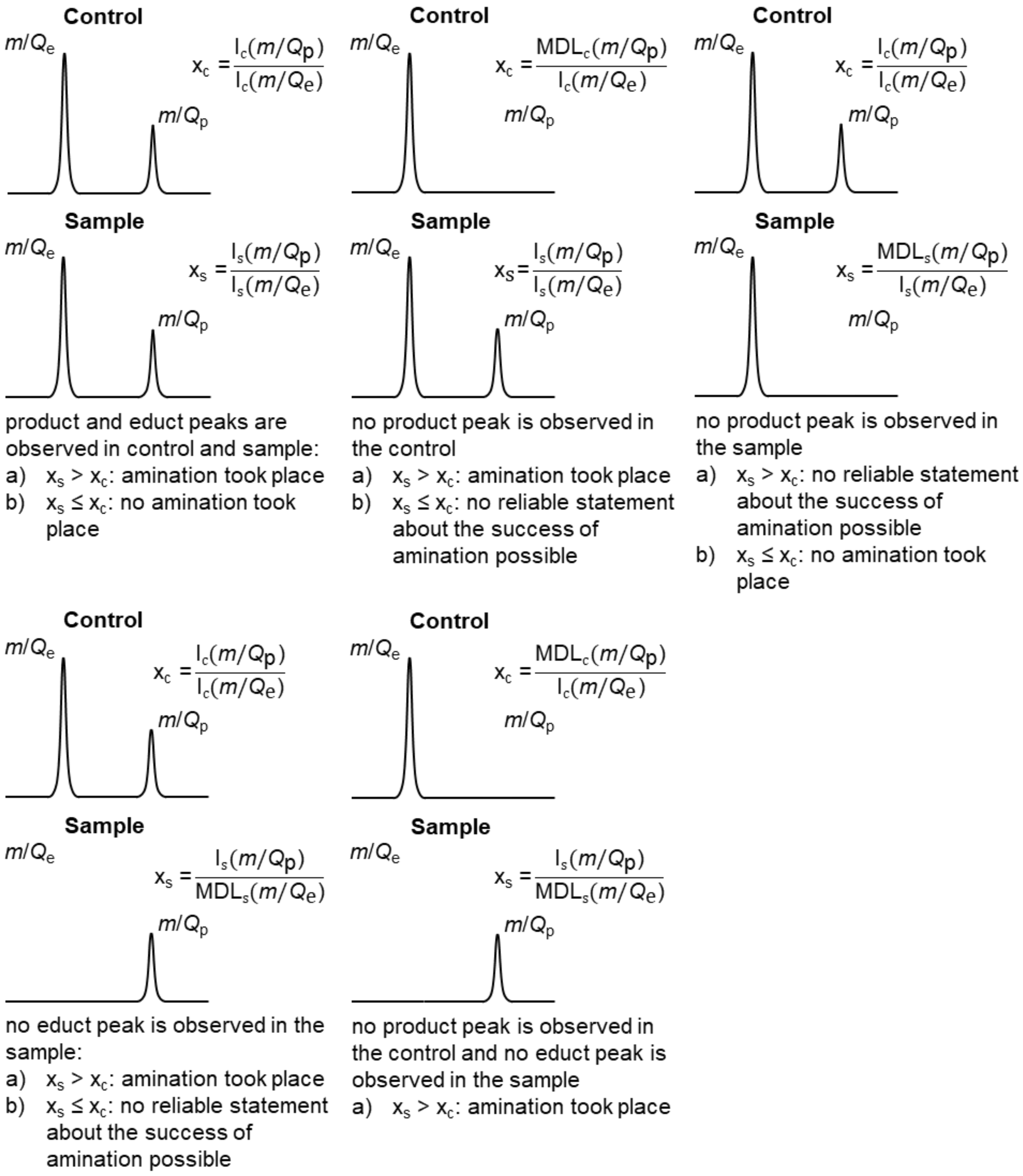

Figure S3 Possible scenarios leading to the detection of ketone-containing molecular species. Cases in which educt and product peaks were absent for the same sample and cases in which educt or product peak were missing for both sample and control were not considered.

If more than one product peak was assigned to a particular educt peak and if all products were reliably detected, we refer these identifications to as “reliably detected combinations”. In contrast, if in these cases at least one product peak was unreliably assigned to a particular educt peak, we refer these identifications to as “unreliably detected combinations”. If not otherwise noted, we only further analyzed “reliably detected combinations”.

S1.8 Selected scenarios leading to potential double counting of peaks identified as products

Scenarios exist that potentially lead to the assignment of product peaks to more than one educt peak. Figure S4 shows two of these scenarios. These cases only appear in a few percent of detections. We have deliberately decided to count these cases twice, as it is usually not possible to clearly define the product/educt relationship. Further, we assume that in these cases it is at least as likely that a potential product peak represents the product of two potential educts than it represents the product of only one educt.

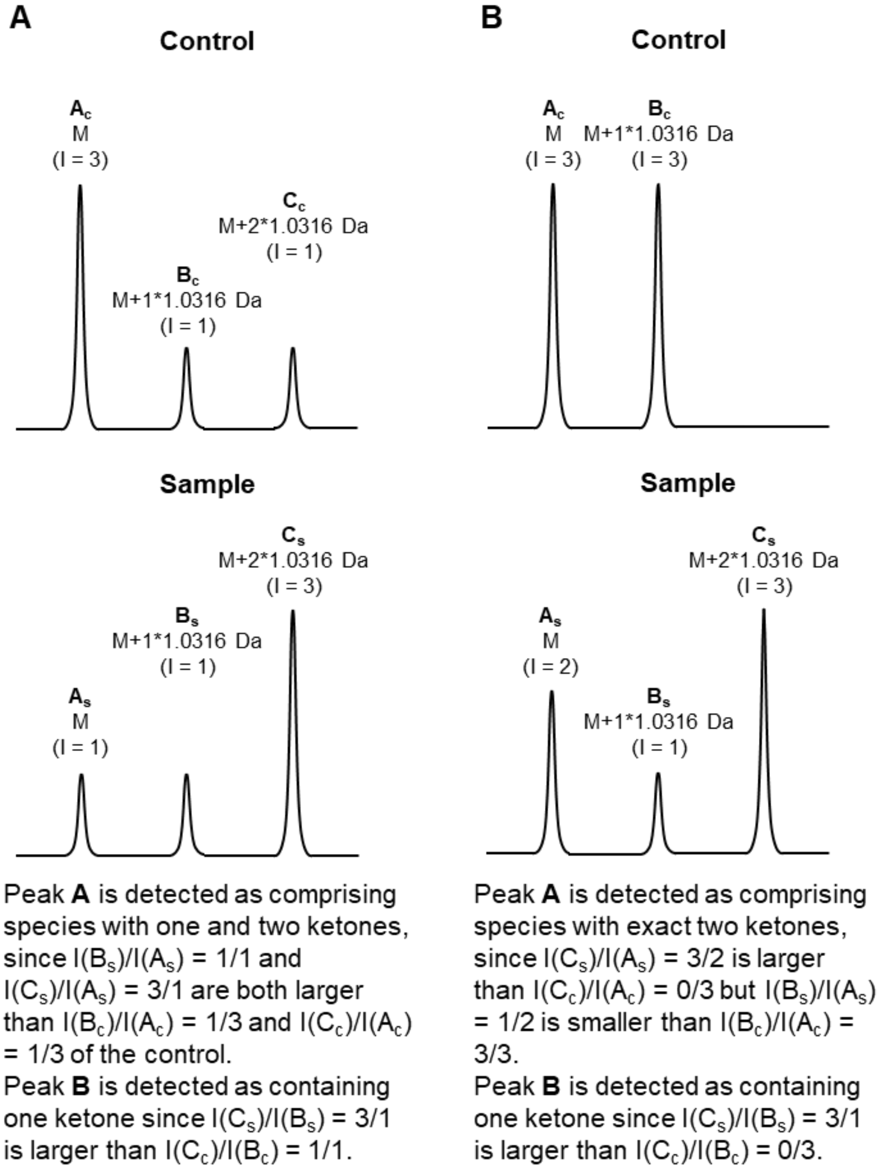

Figure S4 Two possible scenarios that lead to the assignment of product peaks to more than one educt.

S1.9 Conversion

The conversion X is defined as the percentage of a reactant that reacted in a chemical reaction and is calculated as follows:

$$X = \frac{n_{educt}(start) - n_{educt}(end)}{n_{educt}(start)} \cdot 100\%$$

with:

$n_{educt}(start)$: amount of educt prior to the reaction, i.e., in the control.

$n_{educt}(end)$: amount of educt left after the reaction, i.e., in the samples that underwent reductive amination.

For calculating the conversion based on mass spectrometry, the intensity of the reactant's molecular ion can be used if all samples were measured at the same concentration:

$$X = \frac{I_{control} - I_{sample}}{I_{control}} \cdot 100\%$$

with:

$I_{control}$: molecular ion intensity of selected educt in the control, resembling the concentration prior to the reaction.

I_{sample}: molecular ion intensity of selected educt in a sample that underwent reductive amination, resembling the concentration after the reaction.

Since mass spectra of samples were not measured at exactly the same concentrations and also no internal standard was used, we assumed in first approximation that the total intensity of mass spectra did not change due to the reaction (i.e., we assumed similar ionization properties of all samples) if samples would have been measured at exactly the same concentrations. This allows to use the total intensity ratio of sample and control to correct for fluctuating sample concentrations and we calculated the conversions X for all reliably detected molecular ions (partially) comprising ketone-containing species as follows:

$$X = \frac{\frac{\sum I_{sample}}{\sum I_{control}} \cdot I_{control} - I_{sample}}{\frac{\sum I_{sample}}{\sum I_{control}} \cdot I_{control}} \cdot 100\%$$

The fact that analytical replicates are subjected to natural fluctuations in intensity can yield to negative conversions. We calculated the conversion only for reliably detected combinations (*cf*. S1.7).

S1.10 Estimation of the molar proportion of ketone-containing compounds

Assuming that all compounds have similar ionization efficiencies, we estimated the molar proportion of ketone-containing species x by summation of the individual molar ratios x_i of molecular ions that were detected as reaction products:

$$x = \sum x_i \cdot 100\%$$

with:

$\sum x_i$: sum of molar ratios of all molecular ions that were identified as reaction products.

The individual molar ratios x_i were calculated as the intensity proportion of a given molecular ion identified as reaction product with respect to the total spectral intensity as follows:

$$x_i = \frac{I_{sample} - \frac{\sum I_{sample}}{\sum I_{control}} \cdot I_{control}}{\sum I_{sample}}$$

with:

$\sum I_{sample}$: total intensity of the mass spectrum of the sample.

$\sum I_{control}$: total intensity of the mass spectrum of the control.

$I_{control}$: molecular ion intensity of selected product in the control, resembling the concentration prior to the reaction.

I_{sample}: molecular ion intensity of selected product in a sample that underwent reductive amination, resembling the concentration after the reaction.

In contrast to the calculation of the conversion, we also considered unreliably detected combinations (*cf*. S1.7) in which at least one product peak was reliably detected for the estimation of the molar ratio. For example, if a product peak representing species with one keto group was unreliably detected but another product peak representing species with two keto groups was reliably detected, no conversion for the respective educt was calculated but the molar ratio for the reliably detected product with two keto groups was calculated.

S2 Supplementary results

S2.1 ^{1}H NMR spectra of isolated products

1,2,3,4-Tetrahydro-1-naphthylamine

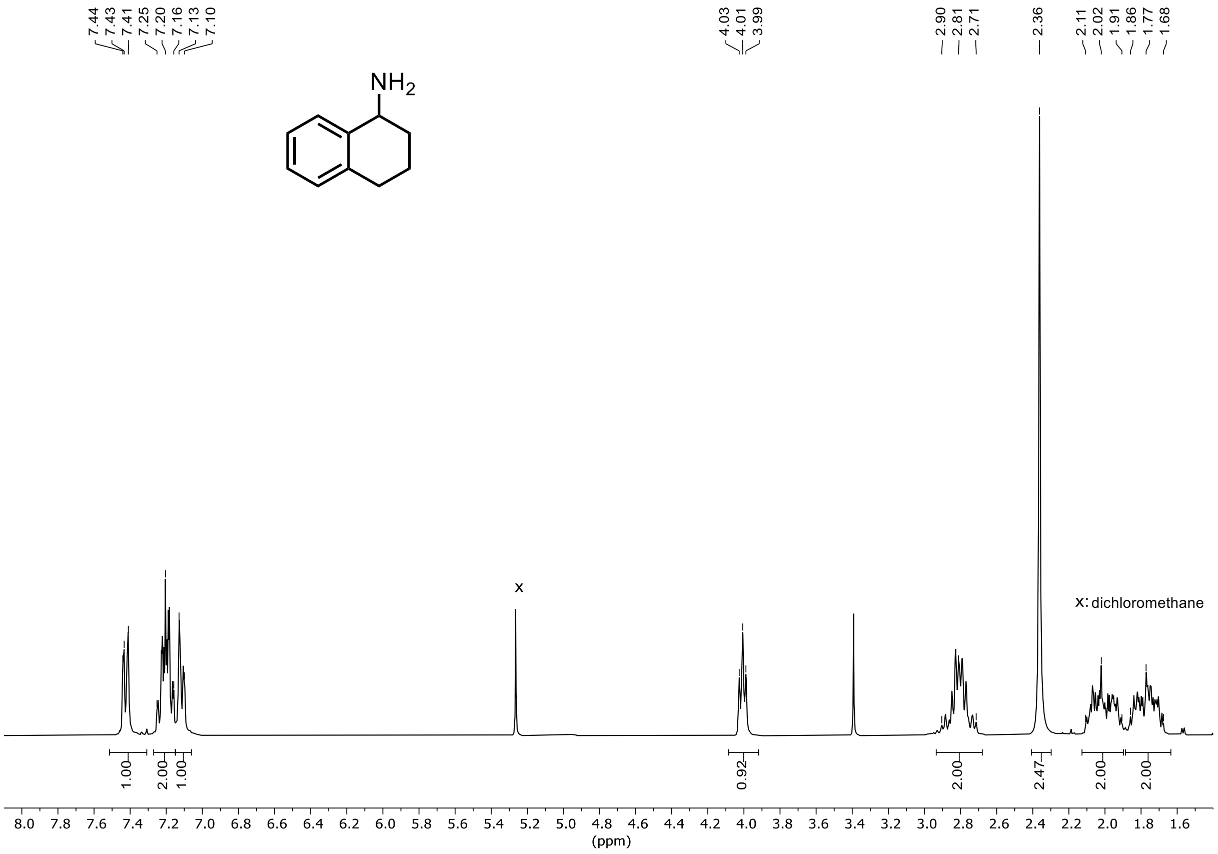

Figure S5 ^{1}H NMR spectrum (300 MHz, $CDCl_3$) of 1,2,3,4-tetrahydro-1-naphthylamine.

^{1}H NMR (300 MHz, $CDCl_3$): δ = 1.68–1.86 (m, 2H), 1.91–2.11 (m, 2H), 2.36 (brs, 2H, NH_2), 2.71–2.90 (m, 2H), 4.01 (t, J = 5.6 Hz, 1H), 7.10–7.13 (m, 1H), 7.16–7.25 (m, 2H), 7.41–7.44 (m, 1H) ppm. All spectroscopic data were in accordance with the literature.[13,14]

1-Methyl-3-phenylpropylamine

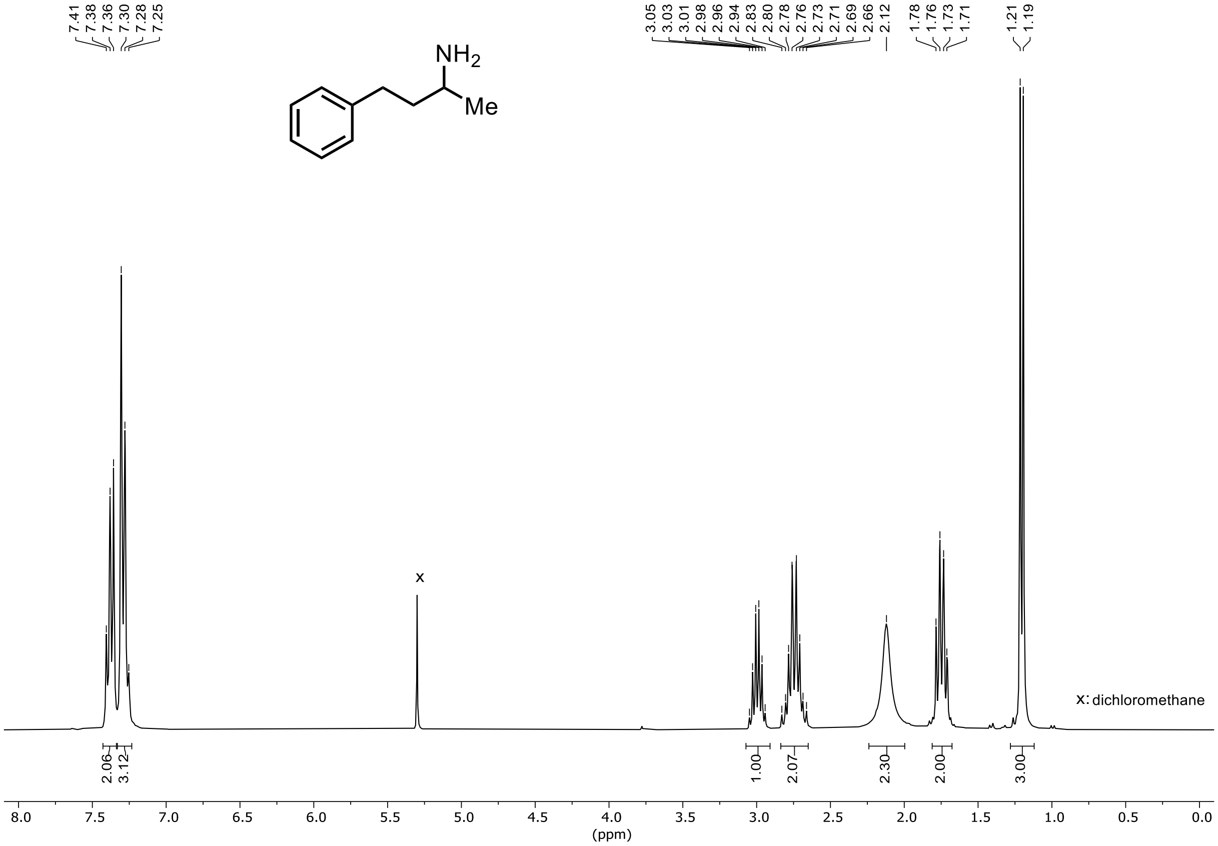

Figure S6 ^{1}H NMR spectrum (300 MHz, $CDCl_3$) of 1-methyl-3-phenylpropylamine.

^{1}H NMR (300 MHz, $CDCl_3$): δ = 1.20 (d, J = 6.3 Hz, 3H), 1.71–1.78 (m, 2H), 2.12 (brs, 2H, NH_2), 2.66–2.83 (m, 2H), 2.94–3.05 (m, 1H), 7.25–7.30 (m, 3H), 7.36–7.41 (m, 2H) ppm. All spectroscopic data were in accordance with the literature.[15]

S2.2 Principal coordinate analysis (PCoA) of the isotopically unlabeled samples

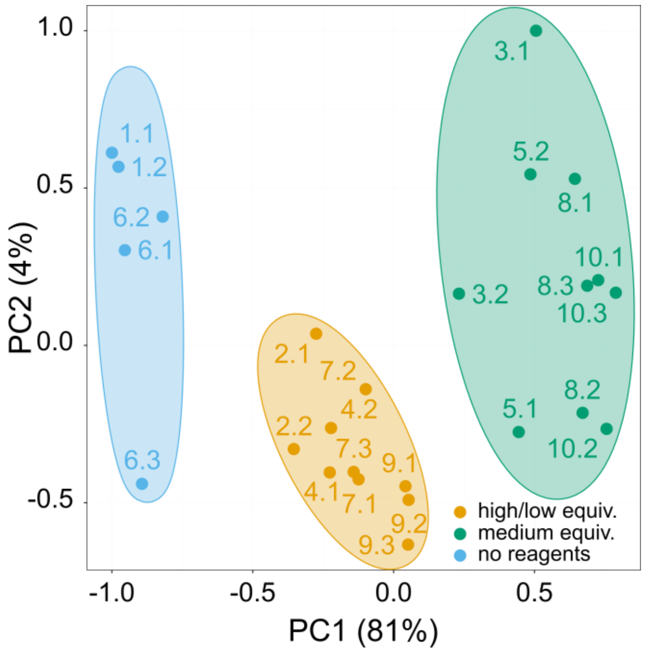

Figure S7 Principal coordinate analysis (PCoA) based on Bray-Curtis dissimilarities calculated from normalized molecular formula intensities of technical replicates. For the labeling of samples we refer to the main manuscript.

S2.3 Selected sections of exemplary mass spectra

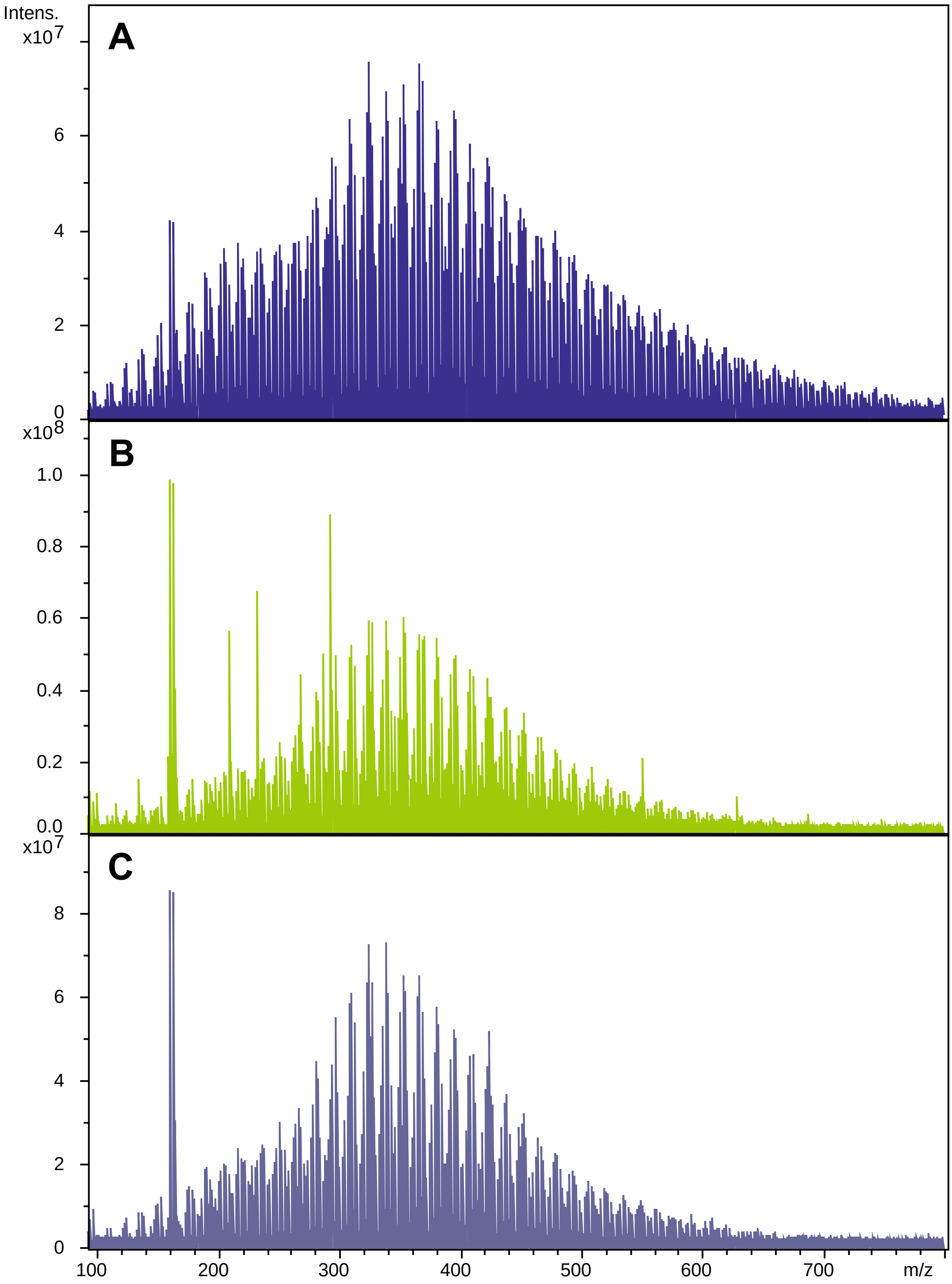

Figure S8 Mass spectral sections from *m*/*z* 92 to *m*/*z* 800 of A) a control (treatment 1, first technical replicate), B) a sample treated with unlabeled NH_4OAc and $NaBH_3CN$ for 48 h (treatment 5, first technical replicate) and C) a sample treated with labeled $^{15}NH_4OAc$ and $NaBH_3CN$ for 48 h (treatment 14, second technical replicate).

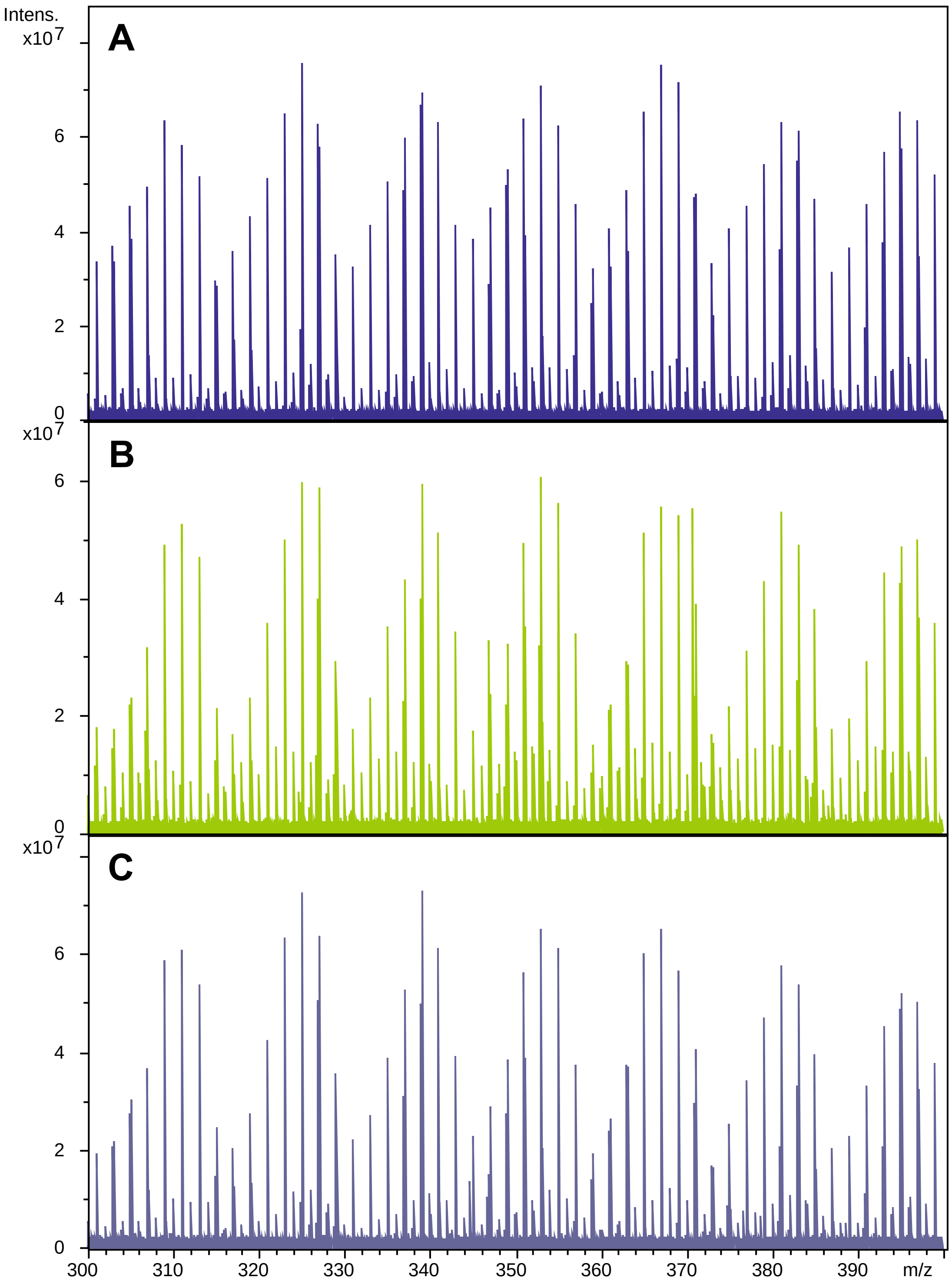

Figure S9 Mass spectral sections from *m*/*z* 300 to *m*/*z* 400 of A) a control (treatment 1, first technical replicate), B) a sample treated with unlabeled NH_4OAc and $NaBH_3CN$ for 48 h (treatment 5, first technical replicate) and C) a sample treated with labeled $^{15}NH_4OAc$ and $NaBH_3CN$ for 48 h (treatment 14, second technical replicate).

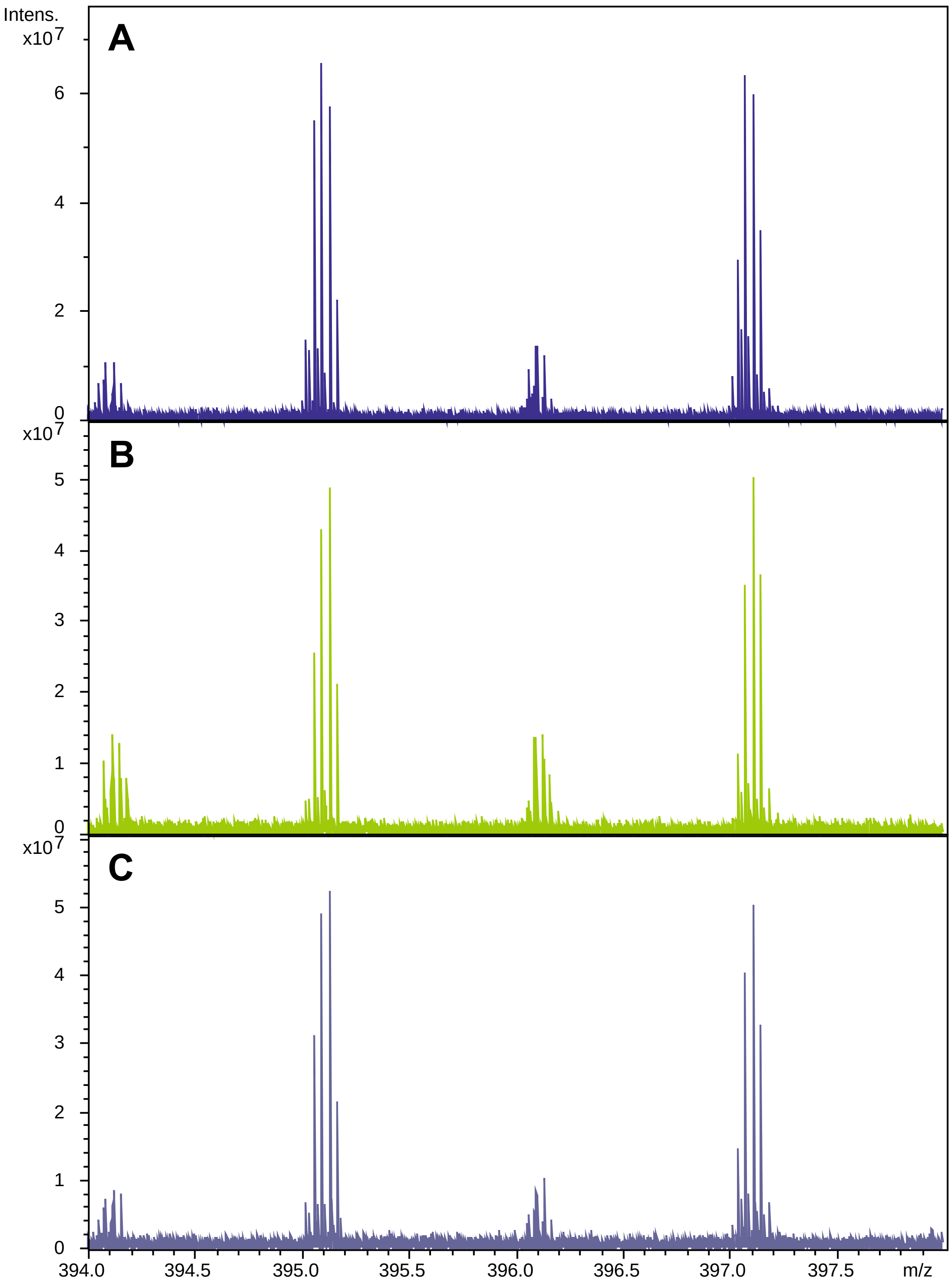

Figure S10 Mass spectral sections from *m*/*z* 394 to *m*/*z* 398 of A) a control (treatment 1, first technical replicate), B) a sample treated with unlabeled NH_4OAc and $NaBH_3CN$ for 48 h (treatment 5, first technical replicate) and C) a sample treated with labeled $^{15}NH_4OAc$ and $NaBH_3CN$ for 48 h (treatment 14, second technical replicate).

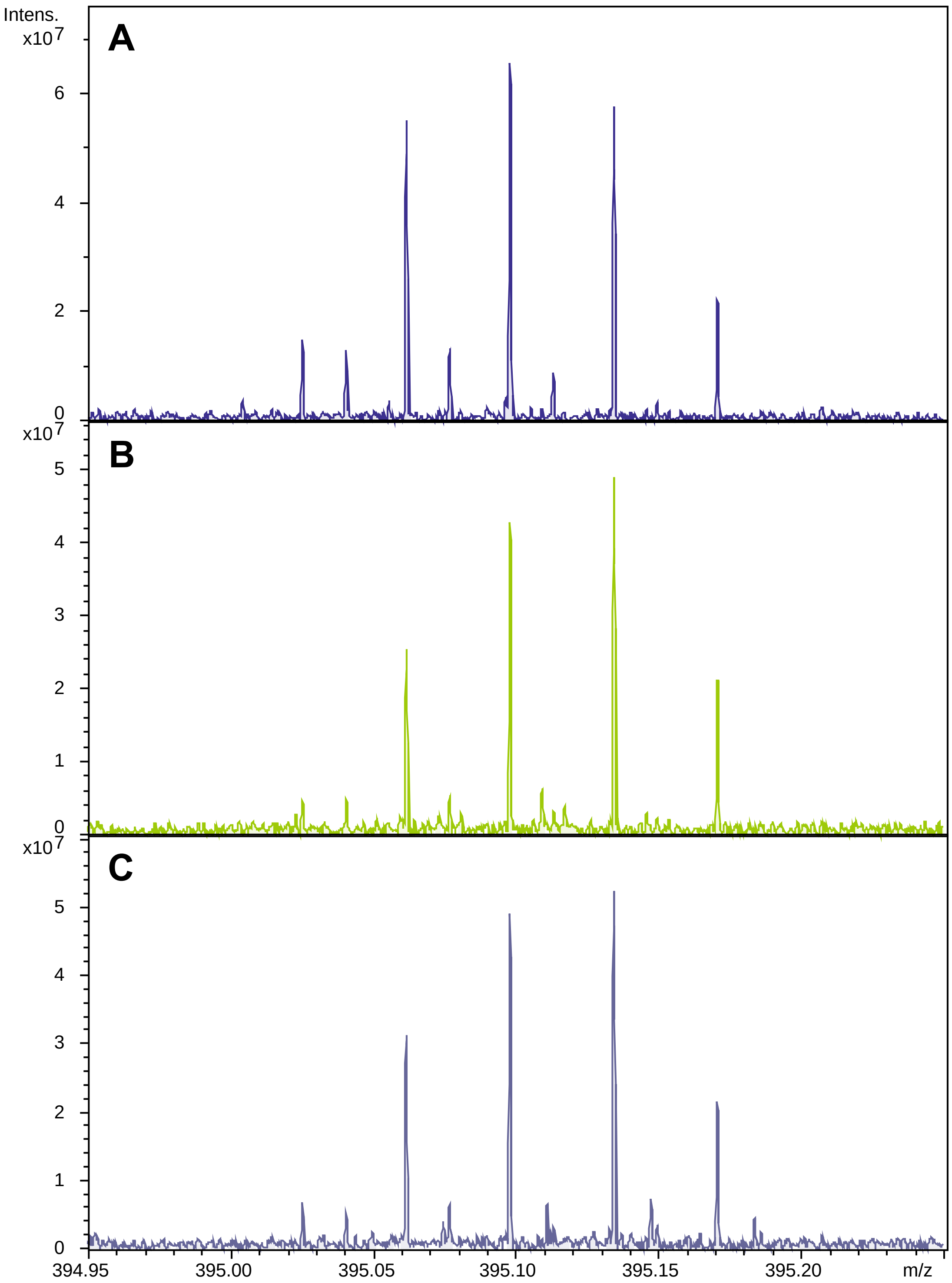

Figure S11 Mass spectral sections from *m*/*z* 394.95 to *m*/*z* 395.25 of A) a control (treatment 1, first technical replicate), B) a sample treated with unlabeled NH_4OAc and $NaBH_3CN$ for 48 h (treatment 5, first technical replicate) and C) a sample treated with labeled $^{15}NH_4OAc$ and $NaBH_3CN$ for 48 h (treatment 14, second technical replicate).

S2.4 Exemplary mass spectra demonstrating the detection of reaction products

Figure S12 displays mass spectral sections illustrating the nominal mass 339 (left panel) alongside sections at nominal masses 340 (middle panel) and 341 (right panel). The latter two represent the nominal masses where potential reaction products may occur, which are indicative for the presence of one keto group in isomers represented by the parent ion at *m*/*z* 339. The two peaks with the highest intensities along with their associated potential products are labeled as 'a' and 'b', respectively. Mass-to-charge ratios and intensity values associated with these peaks are displayed in Table S4 and the intensity ratios of product and educt in Table S5. For reliable detection, the intensity ratio of the product and educt derived from the mass spectra of the derivatized samples must exceed the same ratio in the controls multiplied by the detection ratio. To exemplify, we assessed peak 'a' using the unlabeled dataset. Whereas the intensity ratio of product and educt for this peak in the unlabeled sample was 0.31, it was 0.05 in the control. The optimized detection ratio for this setup was 1.7. Since 0.31 is larger than 0.05 multiplied by 1.7, our routine identified a positive detection. In the example shown in Figure S11, we observed positive detections for both peaks in both the unlabeled (where we searched for the unlabeled mass difference of 1.0316 Da) and the labeled (where we searched for the labeled difference of 2.0287 Da) samples. While the product peak for the product-educt combination 'b' was absent in the unlabeled samples prior to the reaction, the product peak 'a' was already present in the unlabeled samples prior to the reaction.

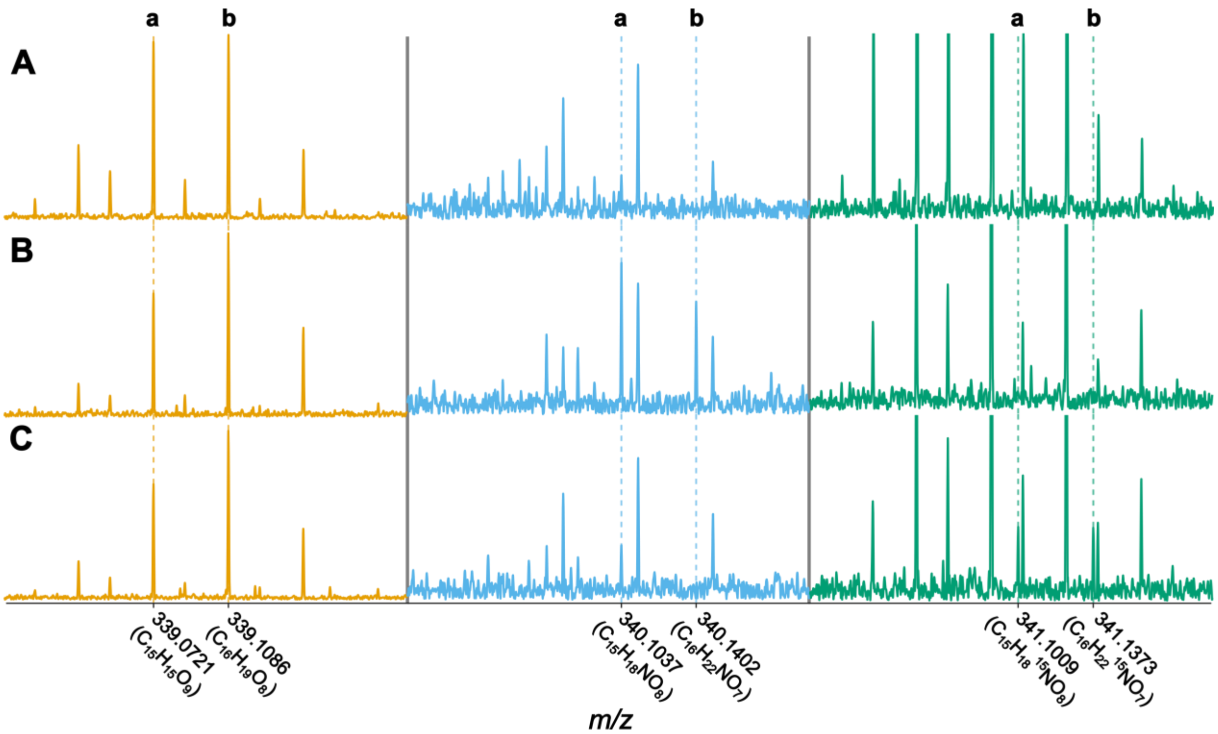

Figure S12 Selected mass spectral sections of SRNOM samples that were treated with A) no reagents (treatment 1, first technical replicate), B) $NH_4OAc/NaBH_3CN$ (treatment 5, first technical replicate) and C) $^{15}NH_4OAc/NaBH_3CN$ (treatment 14, second technical replicate). The lowercase letters refer to selected product-educt combinations.

Table S4 Selected product-educt combinations and their associated intensities as ion counts. Intensities were derived from one randomly chosen analytical replicate and directly taken from Data Analysis.

Dataset	M (educt)		M+1.0316 (unlabeled product)		M+2.0287 (^{15}N-labeled product)	
	m/z	I*10^7	*m/z*	I*10^6	*m/z*	I*10^6
**A** (control)	339.0721	6.73	340.1037	3.57	341.1009	n/a
**A** (control)	339.1086	7.06	340.1402	1.29	341.1373	n/a
**B** (unlabeled)	339.0721	4.03	340.1037	12.3	341.1009	n/a
**B** (unlabeled)	339.1086	5.97	340.1402	9.16	341.1373	1.46
**C** (^{15}N-labeled)	339.0721	4.92	340.1037	4.43	341.1009	5.96
**C** (^{15}N-labeled)	339.1086	7.34	340.1402	1.39	341.1373	5.99

Table S5 Selected peaks (product-educt combinations) and their associated intensity ratios of product and educt, calculated from Table S4.

**Peaks**	**I(M)/I(M+1)** (control)	**I(M)/I(M+1)** (unlabeled)	**I(M)/I(M+1**) (^{15}N-labeled)	**I(M)/I(M+2)** (control)	**I(M)/I(M+2)** (unlabeled)	**I(M)/I(M+2)** (^{15}N-labeled)
**a**	0.05	0.31	0.09	0.00	0.00	0.12
**b**	0.02	0.15	0.02	0.00	0.02	0.08

I(M): molecular ion intensity of the educt.
I(M+1): molecular ion intensity of the product, detected in a difference of 1.0316 Da to the educt peak.
I(M+2): molecular ion intensity of the product, detected in a difference of 2.0287 Da to the educt peak.

S2.5 *Exemplary mass spectra demonstrating the facilitated detection of an increase in peak intensity compared to the detection of a new peak*

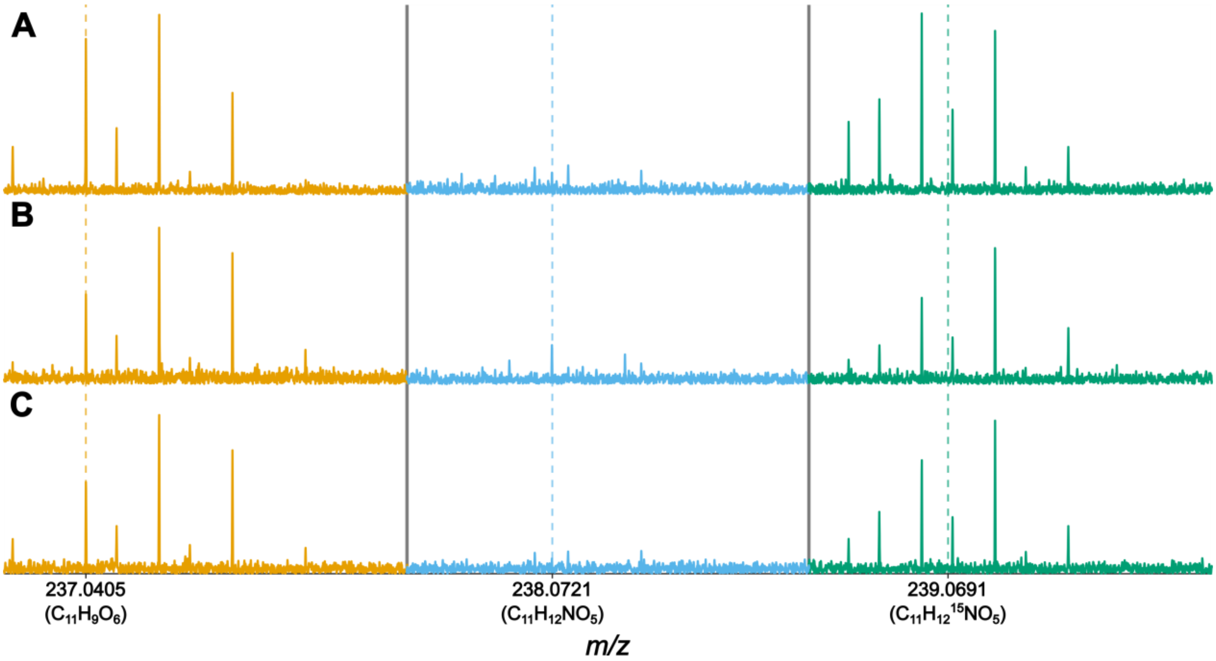

Figure S13 Selected mass spectral sections of SRNOM samples that were treated with A) no reagents (treatment 1, first technical replicate), B) $NH_4OAc/NaBH_3CN$ (treatment 5, first technical replicate) and C) $^{15}NH_4OAc/NaBH_3CN$ (treatment 14, second technical replicate). The isotopically unlabeled reaction product (middle panels, dashed blue line) is easily detected, because a peak with the respective monoisotopic mass has already been present prior to the reaction. The isotopically labeled reaction product (right panels, dashed green line) is not detected, because the corresponding product peak was not present prior to the reaction.

S2.6 Detected ketones

Table S6 Detected molecular ions containing a reliably detected combination of up to three keto groups and their mean conversion for experiments with a reaction time of 48 h using unlabeled NH_4OAc.

#	**1.1 as control, optimized *dr* = 1.7**		**1.2 as control, optimized *dr* = 1.6**		**total peaks**
	detected peaks	conversion [%]	detected peaks	conversion [%]	
**1.1**	0	n/a	9	8	4668
**1.2**	11	14	0	n/a	4465
**2.1**	858	20	871	20	4666
**2.2**	762	22	784	22	5011
**3.1**	685	11	692	13	2435
**3.2**	1203	29	1209	29	4447
**4.1**	858	17	871	17	4204
**4.2**	850	17	858	17	4167
**5.1**	1099	29	1099	30	3836
**5.2**	896	19	901	19	2971

Table S7 Detected molecular ions containing a reliably detected combination of up to three keto groups and their mean conversion for experiments with a reaction time of 168 h using unlabeled NH_4OAc.

#	**6.1 as control, optimized *dr* = 1.8**		**6.2 as control, optimized *dr* = 1.5**		**6.3 as control, optimized *dr* = 1.7**		**total peaks**
	detected peaks	conversion [%]	detected peaks	conversion [%]	detected peaks	conversion [%]	
**6.1**	0	n/a	1	19	4	35	4716
**6.2**	16	-5	0	n/a	11	-23	2305
**6.3**	23	41	0	n/a	0	n/a	5067
**7.1**	917	20	340	32	917	16	4223
**7.2**	909	23	285	33	927	18	4829
**7.3**	816	23	237	32	856	19	4875
**8.1**	1282	33	814	44	1297	29	4105
**8.2**	1398	38	687	48	1429	33	4919
**8.3**	1220	34	781	45	1216	29	4001
**9.1**	827	16	448	31	811	13	3605
**9.2**	1038	21	426	33	1053	17	4274
**9.3**	1068	25	370	35	1109	20	4657
**10.1**	1145	31	795	43	1155	26	3640
**10.2**	1348	38	858	49	1359	35	4212
**10.3**	1020	27	828	43	1005	23	3076

Table S8 Detected molecular ions containing a reliably detected combination of up to three keto groups and their mean conversion for experiments using isotopically labeled $^{15}NH_4OAc$ without the addition of molecular sieve.

#	**11.1 as control, optimized *dr* = 1.5**		**11.2 as control, optimized *dr* = 1.5**		**11.3 as control, optimized *dr* = 1.4**		**total peaks**
	detected peaks	conversion [%]	detected peaks	conversion [%]	detected peaks	conversion [%]	
**11.1**	0	n/a	7	52	4	33	4223
**11.2**	7	29	0	n/a	6	46	4454
**11.3**	7	46	7	61	0	n/a	4251
**12.1**	773	19	792	17	781	19	4394
**12.2**	819	20	837	18	821	21	4676
**12.3**	749	17	767	16	751	17	4342

Table S9 Detected molecular ions containing a reliably detected combination of up to three keto groups and their mean conversion for experiments using isotopically labeled $^{15}NH_4OAc$ with the addition of molecular sieve.

#	**13.1 as control, optimized *dr* = 1.2**		**13.2 as control, optimized *dr* = 1.2**		**13.3 as control, optimized *dr* = 1.5**		**total peaks**
	detected peaks	conversion [%]	detected peaks	conversion [%]	detected peaks	conversion [%]	
**13.1**	0	n/a	6	35	6	34	2928
**13.2**	7	40	0	n/a	3	41	3230
**13.3**	4	84	2	70	0	n/a	3890
**14.1**	281	1	284	-3	285	-8	2412
**14.2**	434	15	463	12	467	11	3400
**14.3**	315	8	321	5	322	0	2811

S2.7 Experimental conversion as a function of O/H values

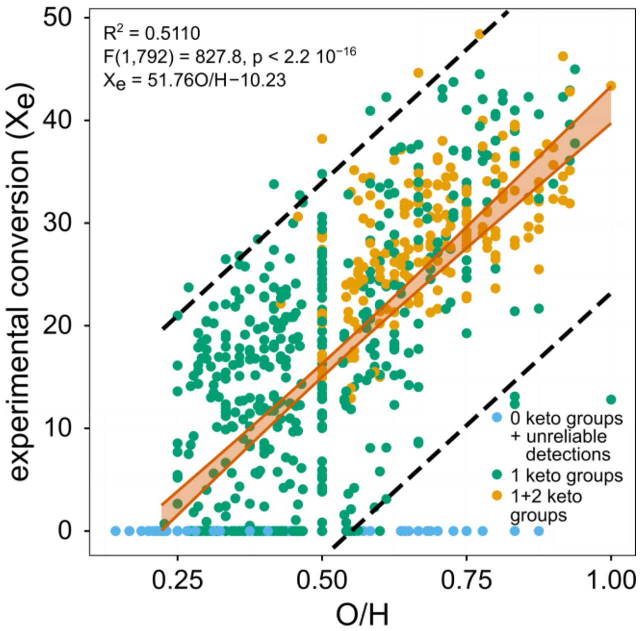

Figure S14 Experimental conversion (X_e) of individual molecular formulas as a function of their O/H values. Each dot represents the conversion of a given molecular formula and was colored in blue for molecular formulas lacking or uncertain to contain ketone moieties, in green for those with exactly one and in orange for those with one or two detected ketone moieties. The experimental conversion was calculated from the intensity ratio of mass peaks between the control (treatment 1, second technical replicate) and treatment 3 (second technical replicate) after formula attribution with ICBM-OCEAN and further processing as described in S1.3. Prior to linear regression, intensity values less than 20% of the maximum detected intensity for the control were removed and negative conversions as well as conversions of compounds unreliably or not identified to contain ketone moieties were set to zero. Confidence (ocher line) and prediction bands (dashed black line) were calculated for a significance level of 0.05.

S2.8 Compound group classification of molecular formulas

ICBM-OCEAN assigns molecular formulas to four descriptive compound groups, namely aromatic (if $AI_{mod} > 0.5$), highly unsaturated (if $AI_{mod} < 0.5$ and $H/C < 1.5$), unsaturated (if $H/C \geq 1.5$ and $H/C \leq 2.0$) and saturated (if DBE = 0) compounds. We investigated the distribution of the two most prominent reliably detected ketone combinations (exactly one keto group detected for a given molecular ion and simultaneously one as well as two keto groups detected for a given molecular ion) within these groups (Figure S15). The combined distributions of molecular formulas (partially) representing isomers containing exactly one keto or one and two keto groups (first bar), respectively, resembled the global distribution across all molecular formulas (last bar). In contrast, molecular formulas representing isomers with exactly one keto group were predominantly detected in highly unsaturated (second bar) and those representing isomers with exactly one and two keto groups predominantly in aromatic structures (third bar).

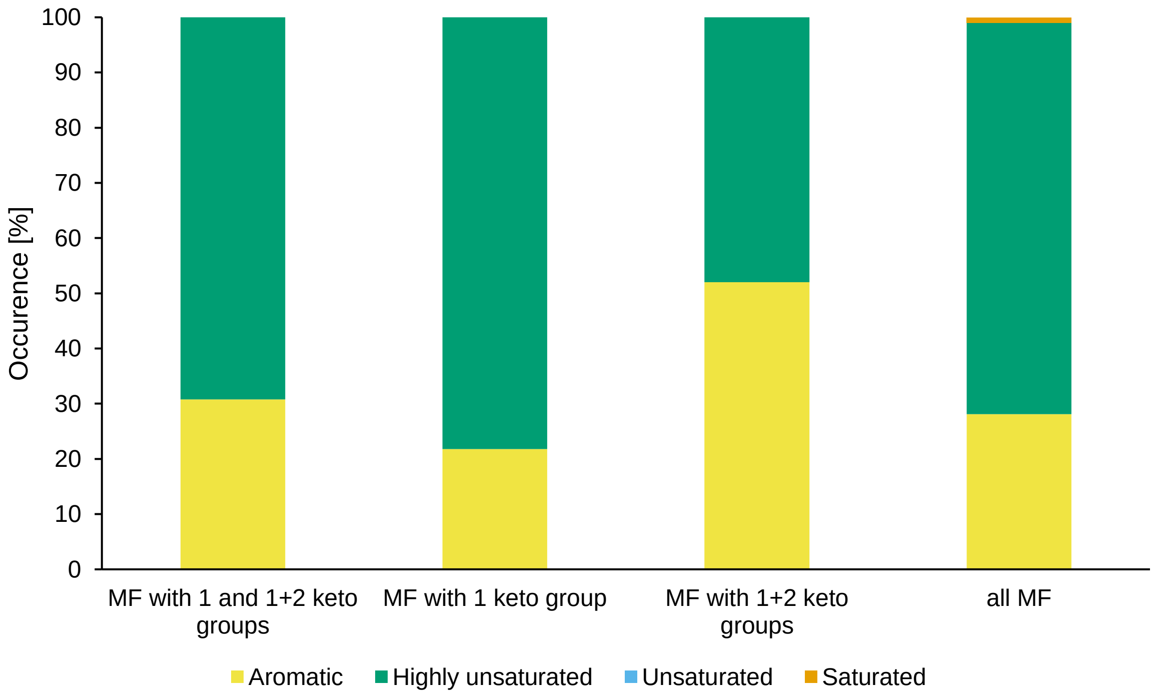

Figure S15 Distribution of the two most prominent reliably detected ketone combinations among compound groups as assigned by ICBM-OCEAN. Displayed is the percentage of molecular formulas assigned to the respective compound classes of treatment 1 (second technical replicate) as derived from the detection in treatment 3 (second technical replicate). MF: molecular formulas.

S2.9 Calculation of the upper oxygen content that is bound in the form of ketone moieties in SRNOM

The upper limit of oxygen bound in the form of ketones in SRNOM was calculated based on published elemental analysis and ^{13}C NMR spectroscopic data. These data were derived for SRNOM (1R101N) from the International Humic Substances Society (IHSS). Please note that we used another batch, 2R101N, in our study, but the respective ^{13}C NMR data were not available from the IHSS. However, both SRNOM batches have similar elemental compositions and we do not expect a large discrepancy in the distribution of functional groups. We calculated the elemental composition in mole percentages of SRNOM from its elemental composition in mass percentages (Table S10). According to the available ^{13}C NMR data (Table S11, please note that these are semi-quantitative solid-state NMR data), 8% of the carbon is bound in the form of carbonyl (including ketones, aldehydes and quinones). Thus, 0.08*38.68% ≈ 3.09% of the amount of substance in SRNOM is bound in carbonyl carbons. The same amount of substance in SRNOM must be bound in oxygen to form ketones, aldehydes or quinones. This implies, that 3.09%/23.63%*100% ≈ 13.1% of the oxygen is bound in the form of carbonyl compounds. Since it is likely that also quinones and aldehydes contribute somewhat to the carbonyl compounds, this estimate constrains the upper limit of oxygen bound in the form of ketones.

Table S10 Elemental composition of SRNOM (1R101N) as derived from the IHSS website.[16]

	C	H	O	N	S	P
**mass percentage**	52.47	4.19	42.69	1.1	0.65	0.02
**mole percentage**	38.68	36.81	23.63	0.70	0.18	0.01

Table S11 Estimates of carbon distribution among structural features determined by semi-quantitative solid-state ^{13}C NMR as derived from the IHSS website.[17]

	Carbonyl	Carboxyl	Aromatic	Acetal	Heteroaliphatic	Aliphatic
**ppm range**	220–190	190–165	165–110	110–90	90–60	60–0
**integral**	8	20	23	7	15	27

S2.10 Identification of primary amines as reaction products by NMR

Since samples were acidified prior to SPE and amines have comparably high pK_a values (~9.5–11.0 for simple alkyl amines), products are expected to exist as protonated amines. As mentioned in the main manuscript, we observed HSQC correlations in the range of 20–45 ppm for the ^{15}N dimension and 7.5–8.5 ppm for the ^{1}H dimension, respectively. Whereas the ^{15}N chemical shift values are indicative for any amines, ^{1}H NMR chemical shifts of protonated amines offer a clearer distinction between primary, secondary and tertiary amines. Primary amines typically have ^{1}H NMR chemical shifts between 7.5–9.0 ppm. In contrast, secondary and tertiary amines have chemical shifts between 9.0–10.5 ppm and >10.5 ppm, respectively.[18,19] Thus, the observed HSQC chemical shift values clearly highlight the formation of primary amines.

S2.11 NMR spectra of SRNOM

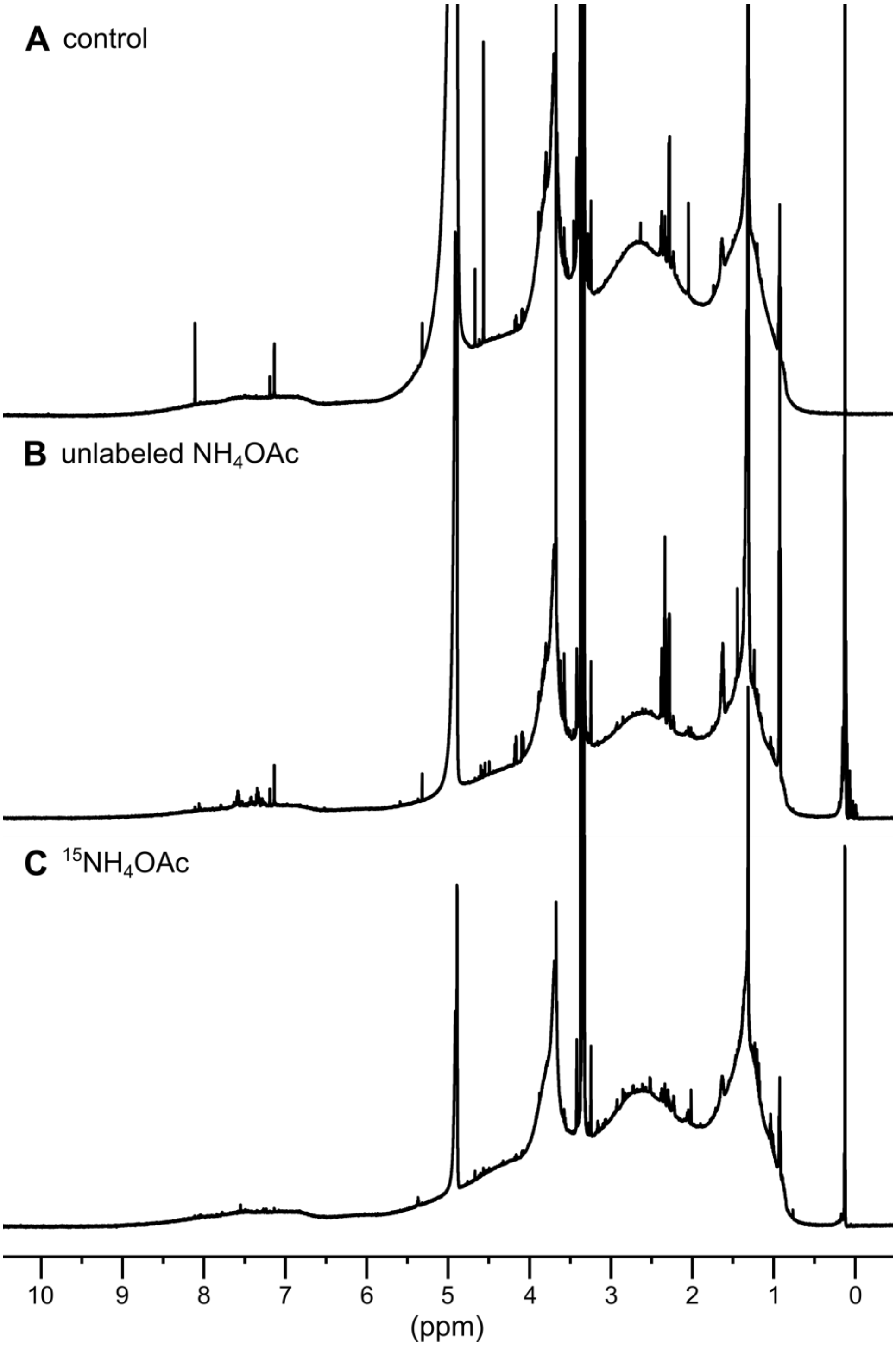

Figure S16 Comparison of 1D ^{1}H NMR spectra of SRNOM treated with A) no reagents (treatment 1, first technical replicate), B) unlabeled $NH_4OAc/NaBH_3CN$ (treatment 3, second technical replicate) and C) $^{15}NH_4OAc/NaBH_3CN$ (treatment 12, second technical replicate). The NMR spectra were recorded at 298 K in CD_3OD using a Bruker Avance Neo 800 MHz (for ^{1}H) instrument equipped with a 5 mm BBO cryoprobe.

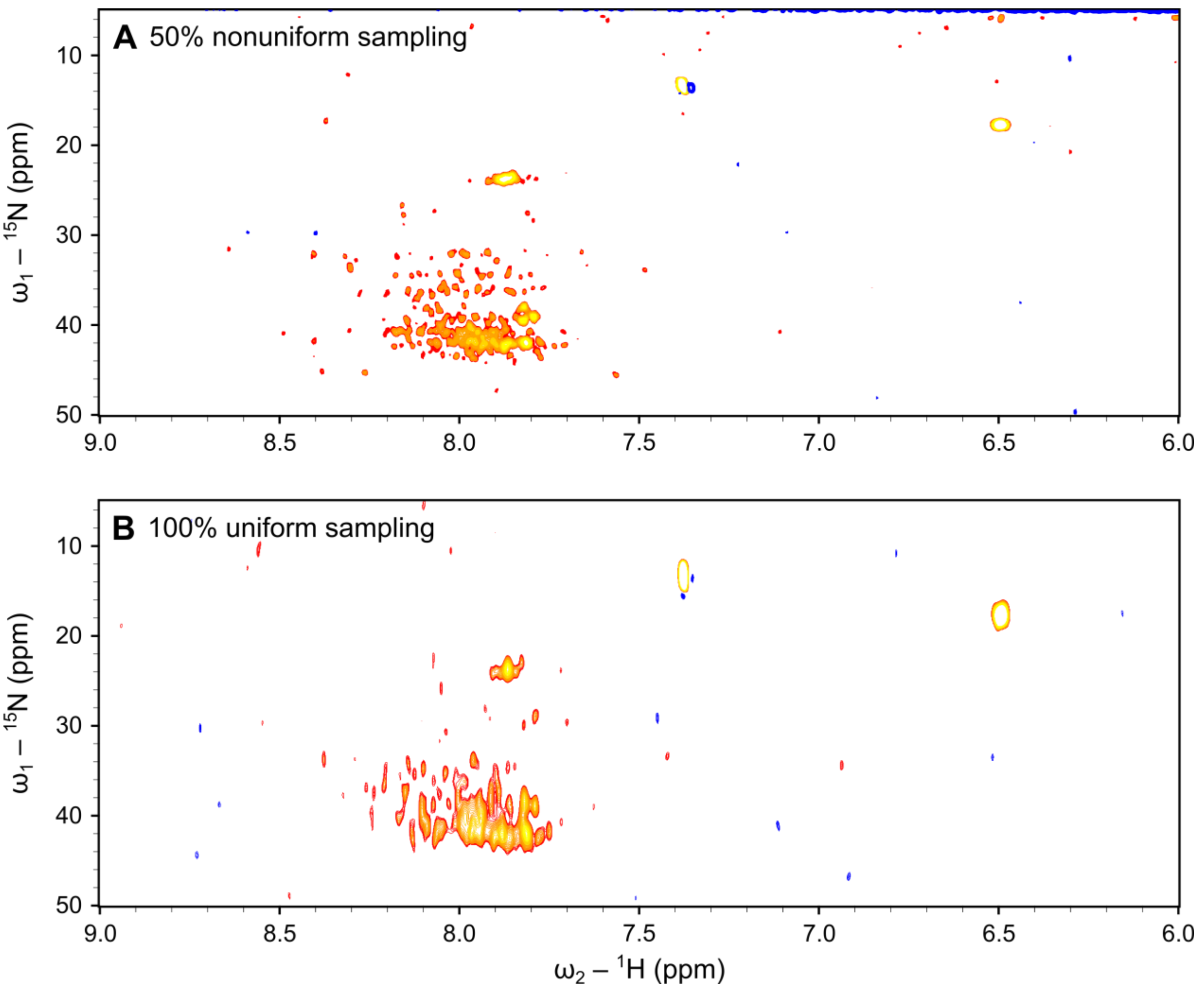

Figure S17 Improving the resolution and detection sensitivity in HSQC using nonuniform sampling. Comparison of 2D $^{1}H,^{15}N$ HSQC spectra of SRNOM treated with $^{15}NH_4OAc/NaBH_3CN$ (treatment 12, second technical replicate) obtained with A) 50% nonuniform sampling and B) 100% uniform sampling. The number of scans per t_1-increment used was 512 and 1400 for 100% uniform and 50% nonuniform sampling, respectively, resulting in experiment times of 39 hours and 53 hours. The increased resolution and detection sensitivity achieved by nonuniform sampling led to a significant increase in the number of detected peaks, from 50 to 88. These additional peaks would have otherwise been buried in the noise level or overlapped with other signals due to the lower resolution in the uniformly sampled spectrum.

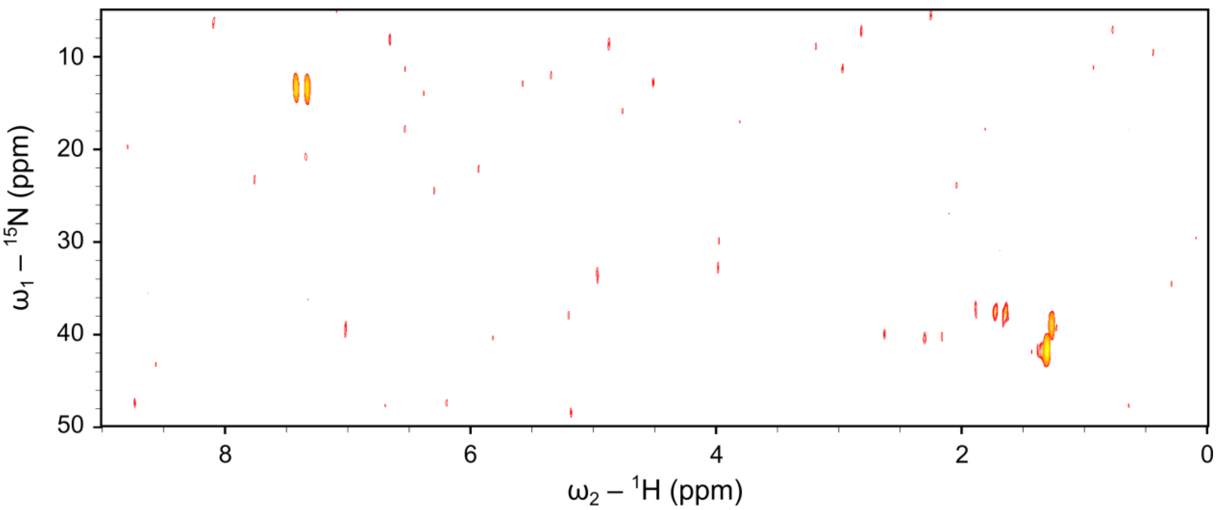

Figure S18 Further 2D NMR evidence for the $^{15}NH_4OAc/NaBH_3CN$ based amination of ketone moieties in SRNOM. 2D ^{1}H,^{15}N heteronuclear multiple bond correlation (HMBC) spectrum of SRNOM treated with $^{15}NH_4OAc/NaBH_3CN$ (treatment 12, second technical replicate). The cross peaks indicate the correlation between nitrogen and proton connected predominantly *via* two and three bonds. Occasionally, one-bond nitrogen-proton correlations also appear in 2D HMBC, as evidenced by the doublet signal around 7.37 ppm in the proton dimension. The same correlation is also detected in the 2D HSQC as a single peak due to the applied nitrogen-proton decoupling during acquisition.

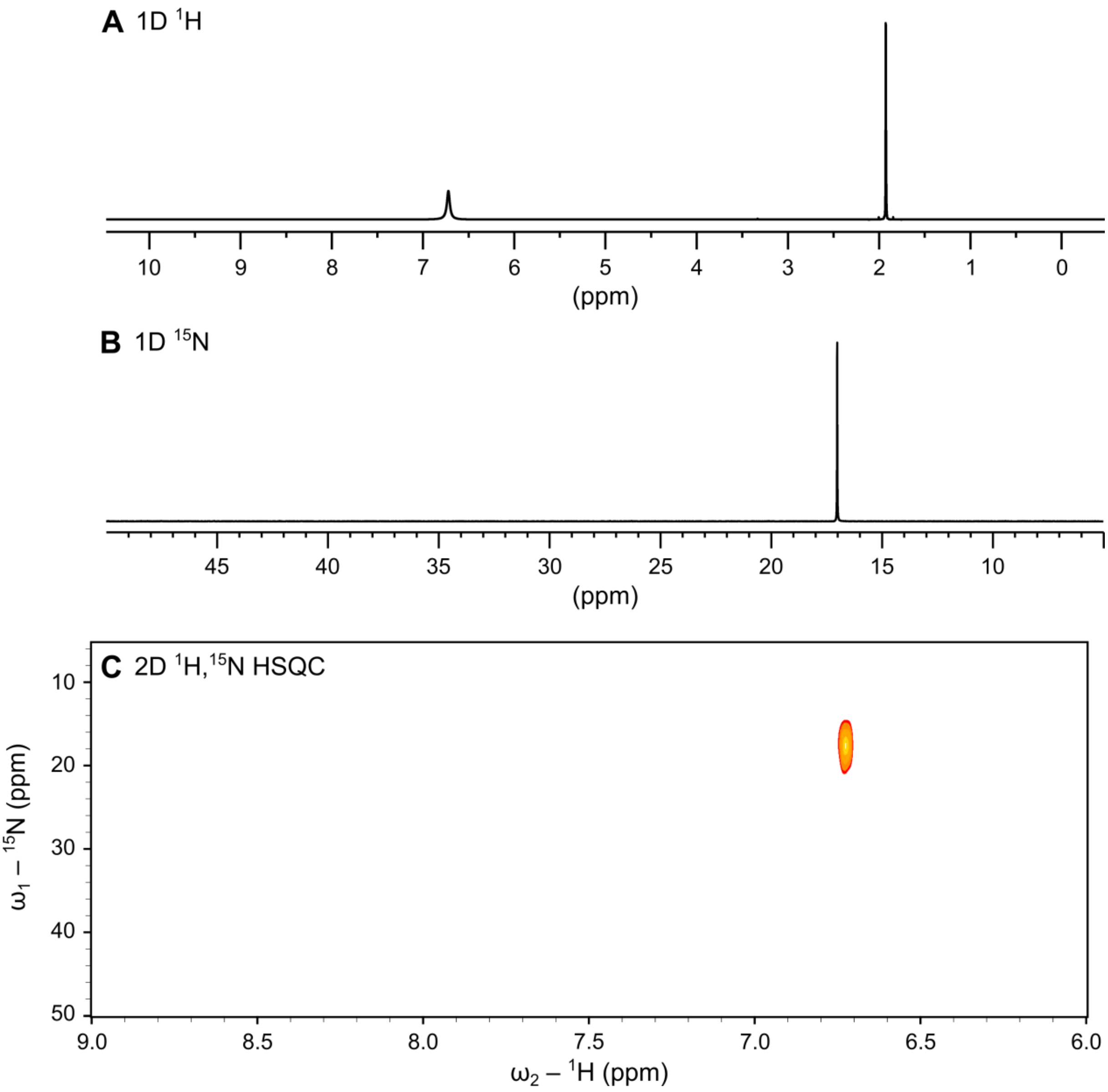

Figure S19 Purity assessment of isotopically labeled $^{15}NH_4OAc$ used for the derivatization of SRNOM. Since the ^1H NMR spectrum (A) displays two expected signals for the CH_3 (1.92 ppm) and NH_4^+ (6.72 ppm) protons and the ^{15}N spectrum shows one signal (17.00 ppm), the reagent is pure and free from impurities. This is further supported by a single nitrogen-proton correlation observed in the 2D $^1H,^{15}N$ HSQC.

References

(1) Dykstra, K. D.; Guo, L.; Birzin, E. T.; Chan, W.; Yang, Y. T.; Hayes, E. C.; DaSilva, C. A.; Pai, L.-Y.; Mosley, R. T.; Kraker, B.; Fitzgerald, P. M. D.; DiNinno, F.; Rohrer, S. P.; Schaeffer, J. M.; Hammond, M. L. Estrogen Receptor Ligands. Part 16: 2-Aryl Indoles as Highly Subtype Selective Ligands for ERα. *Bioorg. Med. Chem. Lett.* **2007**, *17* (8), 2322–2328. https://doi.org/10.1016/j.bmcl.2007.01.054.

(2) Manning, S. J.; Bogen, W.; Kelly, L. A. Synthesis, Characterization, and Photophysical Study of Fluorescent N-Substituted Benzo[Ghi]Perylene "Swallow Tail" Monoimides. *J. Org. Chem.* **2011**, *76* (15), 6007–6013. https://doi.org/10.1021/jo200529p.

(3) Borch, R. F.; Bernstein, M. D.; Durst, H. D. The Cyanohydridoborate Anion as a Selective Reducing Agent. *J. Am. Chem. Soc.* **1971**, *93* (12), 2897–2904. https://doi.org/10.1021/ja00741a013.

(4) Bowles, P.; Clayden, J.; Helliwell, M.; McCarthy, C.; Tomkinson, M.; Westlund, N. Atroposelectivity in the Reactions of Ortholithiated Aromatic Tertiaryamides with Aldehydes. *J. Chem. Soc. Perkin Trans. 1* **1997**, 2607–2616. https://doi.org/10.1039/A701671i.

(5) Laufer, R.; Ng, G.; Liu, Y.; Patel, N. K. B.; Edwards, L. G.; Lang, Y.; Li, S.-W.; Feher, M.; Awrey, D. E.; Leung, G.; Beletskaya, I.; Plotnikova, O.; Mason, J. M.; Hodgson, R.; Wei, X.; Mao, G.; Luo, X.; Huang, P.; Green, E.; Kiarash, R.; Lin, D.-C.-C.; Harris-Brandts, M.; Ban, F.; Nadeem, V.; Mak, T. W.; Pan, G. J.; Qiu, W.; Chirgadze, N. Y.; Pauls, H. W. Discovery of Inhibitors of the Mitotic Kinase TTK Based on N-(3-(3-Sulfamoylphenyl)-1H-Indazol-5-Yl)-Acetamides and Carboxamides. *Bioorg. Med. Chem.* **2014**, *22* (17), 4968–4997. https://doi.org/10.1016/j.bmc.2014.06.027.

(6) Guzii, A. G.; Makarieva, T. N.; Denisenko, V. A.; Dmitrenok, P. S.; Kuzmich, A. S.; Dyshlovoy, S. A.; Von Amsberg, G.; Krasokhin, V. B.; Stonik, V. A. Melonoside A: An ω-Glycosylated Fatty Acid Amide from the Far Eastern Marine Sponge Melonanchora Kobjakovae. *Org. Lett.* **2016**, *18* (14), 3478–3481. https://doi.org/https://doi.org/10.1021/acs.orglett.6b01678.

(7) Merder, J.; Freund, J. A.; Feudel, U.; Hansen, C. T.; Hawkes, J. A.; Jacob, B.; Klaproth, K.; Niggemann, J.; Noriega-Ortega, B. E.; Osterholz, H.; Rossel, P. E.; Seidel, M.; Singer, G.; Stubbins, A.; Waska, H.; Dittmar, T. ICBM-OCEAN: Processing Ultrahigh-Resolution Mass Spectrometry Data of Complex Molecular Mixtures. *Anal. Chem.* **2020**, *92* (10), 6832–6838. https://doi.org/10.1021/acs.analchem.9b05659.

(8) Kazimierczuk, K.; Zawadzka, A.; Koźmiński, W. Optimization of Random Time Domain Sampling in Multidimensional NMR. *J. Magn. Reson.* **2008**, *192*, 123–130. https://doi.org/10.1016/j.jmr.2008.02.003.

(9) Hyberts, S. G.; Takeuchi, K.; Wagner, G. Poisson-Gap Sampling and Forward Maximum Entropy Reconstruction for Enhancing the Resolution and Sensitivity of Protein NMR Data. *J. Am. Chem. Soc.* **2010**, *132* (7), 2145–2147. https://doi.org/10.1021/ja908004w.

(10) G. Wagner. *Schedule Generator Version 3.0*. http://gwagner.med.harvard.edu/intranet/hmsIST/gensched_new.html (accessed 2024-06-19).

(11) Kazimierczuk, K.; Orekhov, V. Y. Accelerated NMR Spectroscopy by Using Compressed Sensing. *Angew. Chemie - Int. Ed.* **2011**, *50* (24), 5556–5559. https://doi.org/10.1002/anie.201100370.

(12) Hyberts, S. G.; Milbradt, A. G.; Wagner, A. B.; Arthanari, H.; Wagner, G. Application of Iterative Soft Thresholding for Fast Reconstruction of NMR Data Non-Uniformly Sampled with Multidimensional Poisson Gap Scheduling. *J. Biomol. NMR* **2012**, *52* (4), 315–327. https://doi.org/10.1007/s10858-012-9611-z.

(13) Bhandari, K.; Srivastava, S.; Shankar, G. Synthesis of Tetrahydronaphthyl Thioureas as Potent Appetite Suppressants. *Bioorg. Med. Chem.* **2004**, *12* (15), 4189–4196. https://doi.org/10.1016/j.bmc.2004.05.039.

(14) Schneider, H.-J.; Agrawal, P. K. Conformations and 13C NMR Shifts in Tetralins. *Org. Magn. Reson.* **1984**, *22* (3), 180–186. https://doi.org/10.1002/mrc.1270220310.

(15) Waser, J.; Nambu, H.; Carreira, E. M. Cobalt-Catalyzed Hydroazidation of Olefins: Convenient Access to Alkyl Azides. *J. Am. Chem. Soc.* **2005**, *127* (23), 8294–8295. https://doi.org/10.1021/ja052164r.

(16) International Humic Substances Society. *Elemental Compositions and Stable Isotopic Ratios of IHSS Samples*. https://humic-substances.org/elemental-compositions-and-stable-isotopic-ratios-of-ihss-samples/ (accessed 2024-06-19).

(17) International Humic Substances Society. *^{13}C NMR Estimates of Carbon Distribution*. https://humic-substances.org/13c-nmr-estimates-of-carbon-distribution-in-ihss-samples/ (accessed 2024-06-19).

(18) Elfinger, M.; Bauer, C.; Schmauch, J.; Moritz, M.; Wichmann, C.; Papp, C.; Kempe, R. General Synthesis of Alkyl Amines via Borrowing Hydrogen and Reductive Amination. *Adv. Synth. Catal.* **2023**, *365* (24), 4654–4661. https://doi.org/https://doi.org/10.1002/adsc.202301179.

(19) Liu, Q.; Zhu, M. Determination of Molar Ratio of Primary Secondary and Tertiary Amines in Polymers by Applying Derivatization and NMR Spectroscopy. *Polym. Test.* **2016**, *56*, 174–179. https://doi.org/https://doi.org/10.1016/j.polymertesting.2016.10.013.
